# TRPM2 knockdown attenuates myocardial apoptosis and promotes autophagy in HFD/STZ-induced diabetic mice via regulating the MEK/ERK and mTORC1 signaling pathway

**DOI:** 10.1007/s11010-024-04926-0

**Published:** 2024-02-03

**Authors:** Feng Hu, Chaoyang Lin

**Affiliations:** Department of Cardiology, Fujian Medical University Union Hospital, Fuzhou, 350001 Fujian China

**Keywords:** Diabetic cardiomyopathy, Transient receptor potential melastatin 2, Apoptosis, Autophagy

## Abstract

Diabetic cardiomyopathy (DCM) is a major complication of diabetes. Transient receptor potential melastatin 2 (TRPM2) activity increases in diabetic oxidative stress state, and it is involved in myocardial damage and repair. We explore the protective effect of TRPM2 knockdown on the progression of DCM. A type 2 diabetes animal model was established in C57BL/6N mice by long-term high-fat diet (HFD) feeding combined with a single injection of 100-mg/kg streptozotocin (STZ). Genetic knockdown of *TRPM2* in heart was accomplished by the intravenous injection via the tail vein of adeno-associated virus type 9 carrying *TRPM2* shRNA. Neonatal rat ventricular myocytes was exposed to 45 mM of high-glucose (HG) stimulation for 72 h in vitro to mimic the in vivo conditions. Western blot, real-time quantitative PCR (RT-qPCR), immunohistochemistry and fluorescence, electron, CCK-8, and flow cytometry were used to evaluate the phenotype of cardiac inflammation, fibrosis, apoptosis, and autophagy. Mice with HFD/STZ-induced diabetes exhibited systolic and diastolic dysfunction, as demonstrated by increased myocardial apoptosis and autophagy inhibition in the heart. Compared to control group, the protein expression of TRPM2, bax, cleaved caspase-3, and P62 was significantly elevated, and the protein expression of bcl-2 and LC3-II was significantly decreased in the myocardial tissues of the HFD/STZ-induced diabetes group. Knockdown of *TRPM2* significantly reversed the HFD/STZ-induced myocardial apoptosis and autophagy inhibition. *TRPM2* silencing attenuated HG-induced apoptosis and autophagy inhibition in primary cardiomyocytes via regulating the MEK/ERK mTORC1 signaling pathway. TRPM2 knockdown attenuates hyperglycemia-induced myocardial apoptosis and promotes autophagy in HFD/STZ-induced diabetic mice or HG-stimulated cardiomyocytes via regulating the MEK/ERK and mTORC1 signaling pathway.

**Supplementary Information:**

The online version contains supplementary material available at 10.1007/s11010-024-04926-0.

## Introduction

The incidence of heart failure is 2 to 5 times greater in diabetic compared with non-diabetic patients independent of age, obesity, dyslipidemia, and coronary artery disease [1–3]. Diabetic cardiomyopathy (DCM) is a separate clinical form of myocardial disease manifested as decreased cardiac performance independent of atherosclerosis, valvular defects, hypertension, and dyslipidemia [4]. Both type 1 diabetes mellitus (T1DM) and type 2 diabetes mellitus (T2DM) could present with DCM [4]. The prevalence of DCM is increasing in parallel with the increase in diabetes mellitus owing to the extension of average life and increased obesity rate, ranging from 19 to 26% [2, 5, 6]. DCM is initially characterized by myocardial fibrosis, dysfunctional remodeling, and associated diastolic dysfunction, later by systolic dysfunction and eventually by clinical heart failure, significantly contributing to the mortality of diabetic patients [4, 7]. However, the exact underlying mechanisms and sequence of DCM remains incompletely understood and effective therapies are limited. The myocardial changes are believed to be induced by hyperglycemia and myocardial insulin resistance [8]. The pathogenesis of myocardial injury is currently recognized to be multifactorial, including increased oxidative stress, lipotoxicity, inflammation, apoptosis, abnormal mitochondrial and cardiomyocyte calcium handling, endoplasmic reticulum stress, and cell signaling impairments [4, 7, 9]. With increasing incidences of diabetes, there is urgent need to understand the pathogenic mechanisms of DCM in order to prevent and treat this devastating disease.

Transient receptor potential melastatin 2 (TRPM2) ubiquitously expressed over the entire body is a unique member of the TRPM channel superfamily comprising a cation channel and a functional enzymatic domain in highly varied C-terminal segments classifying them as chanzymes [10–13]. TRPM2 is widely distributed in the central nervous system, heart, bone marrow, kidney, lung, liver, pancreas, vasculature, and hematopoietic cells [12, 14]. TRPM2 is present in the tetrameric form located in intracellular vesicles and the plasma membrane, with each subunit consisting of six transmembrane segments [13]. TRPM2 channel is permeable primarily to Ca^2+^, Na^+^, and K^+^, and activated by intracellular free adenosine diphosphate ribose (ADP) produced by oxidative stress [11, 13, 15, 16].

The two-in-one protein structure, ubiquitous distribution throughout the body, and redox-sensitive Ca^2+^-permeable cation channel enabled the involvement of TRPM2 in numerous pathophysiological processes, such as oxidative stress, apoptosis and necrosis, pulmonary inflammation, Alzheimer’s disease, ischemic stroke, hypoxic-ischemic brain damage, neurovascular injury, renal inflammation and fibrosis, pancreatitis, liver damage, ischemia–reperfusion injury (IRI), diabetes mellitus, and tumorigenesis [12, 15, 17–44]. TRP channels were highly sensitive to activation by oxidative stress-related diseases, such as Alzheimer’s disease, IRI, and diabetes [45]. At present, it is still controversial whether TRPM2 channel could protect or aggravate myocardial IRI. On the one hand, TRPM2 conferred protection against IRI by reducing production and enhancing scavenging of reactive oxygen species (ROS), thereby reducing IRI-induced oxidative stress [32]. TRPM2-mediated Ca^2+^ influx was necessary to maintain mitochondrial function and protect hearts from IRI and doxorubicin-related cardiotoxicity [33]. For another, accumulation of neutrophils in the reperfused area mediated by TRPM2 activation was implicated in the exacerbation of myocardial IRI [41]. The size of myocardial infarction, adhesion of neutrophils to endothelial cells, and cardiac contractile function were improved in TRPM2-knockout mice subjected to the ligation of the left main coronary artery followed by reperfusion compared to wild-type mice [41]. These discrepancies above likely depend on the relative contributions of the channel vs. the kinase domain and highlight the complexity of the system [46]. These results provided novel insight into the key role of TRPM2 in unexplained cardiomyopathy including DCM.

Knockout of TRPM2 impaired incretins-induced insulin secretion and glucose metabolisms in mice [44]. Pharmacological inhibition of TRPM2 reduced hyperglycemia-induced cognitive impairment by down-regulating calcium-related downstream signaling in rats [43]. Diabetes reinforced oxidative stress-induced TRPM2-mediated Ca^2+^ influx and its control by N-acetylcysteine in rat dorsal root ganglion and brain [36]. In consideration of the vital role of TRPM2 in cardiovascular disease and diabetes, we propose the potential of targeting TRPM2 channel as a novel therapeutic strategy for DCM.

## Materials and methods

### Ethics statements

Male C57BL/6N mice obtained from Hunan SJA Laboratory Animal Co., Ltd (Hunan, China) were housed conventionally at 22 ± 2.0 °C and 50% ± 5% humidity under a 12-h light–dark cycle with free access to water and food. Treatment groups were assigned in a randomized fashion. All animal experiments were conducted in compliance with the National Institutes of Health (NIH) policies in the Guide for the Care and Use of Laboratory Animals and were approved by the Animal Care and Use Committee of Fujian Medical University Union Hospital.

### Recombinant adeno-associated virus (AAV) construction and infection of mice

The recombinant AAV9-U6-negative control (NC) and recombinant AAV9-U6-shTRPM2 constructs were constructed and ordered from GeneChem (Shanghai, China), and the titers were ~ 3 × 10^12^ viral genomes per ml (vg/ml). An shRNA sequence targeting TRPM2 (5′-tAACCTTAGCTCATGGATTC CCtcaagagGGGAATCCATGAGCTAAGGTTttttttc-3′), which corresponded to coding regions 68–89 relative to the first nucleotide of the start codon of mouse TRPM2 (GenBank no. NM_138301) and a scrambled (5′-tAATTCTCCGAACGTGTCACGTtcaagagACGTGACACGTTCGGAGAATTttttttc-3′) sequence were cloned into the U6-MCS-CAG-mCherry vector (plasmid GV480).

We knocked down the TRPM2 gene with a single caudal vein injection of ~ 1 × 10^11^ genome copies of recombinant AAV9-U6-shTRPM2 at 6 weeks of age in the corresponding mice. Two weeks later, 24 8-week-old male mice were randomly assigned into four groups: (i) AAV9-U6-NC recombinant-treated control mice (TRPM2^KDNC^; Control group, *n* = 6); (ii) AAV9-U6-NC recombinant-treated mice with HFD/STZ-induced diabetes (TRPM2^KDNC^; Diabetes group, *n* = 6); (iii) AAV9-U6-shTRPM2 recombinant-treated mice (TRPM2^KD^; Control group, *n* = 6); and (iv) AAV9-U6-shTRPM2 recombinant-treated mice with HFD/STZ-induced diabetes (TRPM2^KD^; Diabetes group, *n* = 6).

### T2DM model

We used a high-fat diet (HFD) and streptozotocin injection model to induce T2DM as previously illustrated [47–53]. 8-week-old male mice were fed either a HFD (60% of calories from fat, #MD12033, Meidisen, China) or normal chow. After 4 weeks, the HFD mice who exhibited insulin resistance received an intraperitoneal (i.p.) injection of streptozotocin (STZ, Sigma) at a single dose of 100 mg/kg dissolved in 100-mM citrate buffer (pH 4.5) to partly destroy islet function and raise glucose levels. The normal chow mice received citrate buffer alone and were processed in parallel with the diabetic mice. All mice were maintained on their respective diets until the end of the study. The fasting blood glucose (FBG) level was measured using a blood glucose monitor (ACCU-CHEK Performa, Roche, USA), and their weights were monitored once per two weeks. Mice with FBG > 11.1 mmol/L were considered diabetic and were used for the further study.

Twenty weeks after STZ treatment, the mice were anesthetized using an i.p. injection of sodium pentobarbital (50 mg/kg), and the adequacy of anesthesia was confirmed by the absence of a reflex response to a foot squeeze. Afterward, the heart was perfused and partial tissues were fixed in 4% paraformaldehyde for pathological analysis. The remaining tissues were freshly flash-frozen in liquid nitrogen for gene and protein expression analysis.

### Echocardiography

Animals were anaesthetized by isoflurane inhalation (2.5%) plus 1 L/min O_2_. Cardiac structure and function were assessed by echocardiography using a Vevo2100 ultrasound imaging system (VisualSonics Inc., Canada) with a 30-MHz linear array ultrasound transducer (MS-400, VisualSonics Inc.). Parasternal long-axis images were acquired in B-mode with appropriate position of the scan head to identify the maximum LV length. In this view, the M-mode cursor was positioned perpendicular to the maximum LV dimension in end diastole and systole, and M-mode images were obtained for measuring wall thickness and chamber dimensions. LV ejection fraction (LVEF) and fractional shortening (LVFS) were calculated automatically. We detected the E′ peak and the A′ peak in the mitral valve orifice using tissue Doppler ultrasound. E′/A′ ratio was calculated as an indirect measure of diastolic function.

### Histological studies

The mice were perfused with cold normal saline and hearts harvested, fixed with 4% paraformaldehyde for 24 h at the room temperature, and embedded in paraffin wax. Serial tissue blocks (4 μm thickness) were stained with hematoxylin and eosin (H&E) for assessment of the myocardial injury and inflammation and with Masson’s trichrome (#G1340, Solarbio, Beijing, China) to detect collagen matrix deposition. Then, the samples were observed with a light microscope (Olympus, Japan). To quantify cardiac fibrosis, semi-quantitative analysis of the tissue staining was performed using Image-Pro plus 6.0 (Media Cybernetics, USA).

### Immunohistochemistry

Immunohistochemistry was performed to detect the protein level of α-SMA (myocardial fibrosis marker) and P62 (Autophagy substrates). Briefly, the hearts were embedded in paraffin, and 4-μm slices were cut from the embedded blocks. After paraffin-embedded cardiac sections were deparaffinized and rehydrated, slides were immersed in EDTA antigen retrieval buffer (pH 8.0) for antigen retrieval. Rehydrated slides were blocked using 3% bovine serum albumin for 30 min and then incubated overnight at 4 °C with α-SMA mouse antibody (#BM0002, Boster, Wuhan, China, 1:200) or P62 rabbit antibody (#ab109012, Abcam, 1:400). After washing with PBS, a secondary goat anti-mouse antibody (#5220-0341, SeraCare Inc., USA, 1:200) or goat anti-rabbit antibody (#5220-0336, SeraCare Inc., USA, 1:200) with avidin–biotin complex/horseradish peroxidase, which catalyze the oxidative precipitation of 3,3-diaminobenzidine (DAB, #2005289, DAKO). The non-immune IgG isotype control was used as negative control and gave negative for all samples.

### Immunofluorescent double-staining

After paraffin-embedded cardiac Sections. (4 μm thick) were deparaffinized and rehydrated, slides were immersed in EDTA antigen retrieval buffer (pH 8.0) for antigen retrieval. Then slides were blocked using 3% bovine serum albumin for 30 min and then incubated overnight at 4 °C with TRPM2 antibody (#PA1-46473, Invitrogen, 1:100). After washing with PBS, slides were correspondingly incubated with the goat anti-mouse secondary antibody (Cy3 conjugate, #SA00009-1, Proteintech, Wuhan, 1:100) at 37 °C for 1 h in the darkroom and washed by PBS for three times/5 min. Subsequently, serial sections were stained with FITC-conjugated wheat germ agglutinin (WGA) (#L4895, Sigma, USA) for measurement of cardiomyocyte size in vivo by myocyte cross-section areas [54]. The cell nuclei were stained with 4′,6-diamidino-2-phenylindole (DAPI, #C0065, Solarbio, Beijing) for 10 min, and all stained sections were viewed by fluorescent microscope (Olympus, Tokyo, Japan).

### Terminal deoxynucleotidyl transferase dUTP nick-end labeling (TUNEL) assay

Apoptosis in heart tissue sections was detected using a TUNEL detection kit (#6432344001, Roche, USA) according to the manufacturer’s protocol. The myocardial cytoskeleton was stained with α-actin (#23660-1-AP, Proteintech, Wuhan, 1:100) for co-localization of myocardial cell and slides were correspondingly incubated with the goat anti-rabbit secondary antibody (FITC conjugate, #SA00003-2, Proteintech, Wuhan, 1:100). The cell nuclei were stained with DAPI and TUNEL-positive cells were observed using a fluorescence microscope (Olympus, Tokyo, Japan).

### Evaluation of electron microscopy

About 1-mm3 heart tissues obtained from left ventricular were immediately prefixed in 2.5% glutaraldehyde for 4 h at 4 °C. After rinsed with PBS, the tissues were fixed in 1% osmium tetroxide for 2 h at room temperature. Next, the tissues were dehydrated and then embedded in Epon 812 overnight. The sections were cut on an ultramicrotome (Leica UC 7, Leica) and double stained with uranyl acetate and lead citrate. Ultrastructural studies were performed to probe for double membrane-bound autophagic vacuoles, a long-established analytic gold standard for autophagy which was observed by transmission electron microscope (TECNAI G2 20 TWIN, FEI).

#### Neonatal rat ventricular myocytes (NRVMs)

Neonatal Sprague Dawley (SD) rats with 1–2 days old were provided by the Laboratory Animal Center of Nanchang University (Nanchang, Jiangxi, China). NRVMs were isolated and cultured as described previously with minor modifications [50–52]. Briefly, Neonatal SD rats were anesthetized with carbon monoxide and then sacrificed. Hearts were excised aseptically from 1- or 2-day-old SD rat pups. The atria were removed, and the ventricles were minced and digested in phosphate-buffered saline (PBS) (PYG0021, Boster, Wuhan) containing 0.08% trypsin (T8150, Solarbio, Beijing) and 0.08% type II collagenase (C6885, Sigma, USA) for 8 to 10 cycles. Ventricles were subsequently centrifuged and suspended in Dulbecco’s modified eagle medium (DMEM, Gibco, Rockville, MD, USA) supplemented with 10% fetal bovine serum (FBS, Gibco, Rockville, MD, USA). To reduce non-myocytes, the dissociated cells were pre-plated in regular culture dishes for 90 min. Then, the resultant suspension of myocytes was plated onto gelatin-coated culture dishes at the desired density and incubated in DMEM containing 20% FBS and 100-µM bromodeoxyuridine (BrdU). After 48-h incubation, cells were grown in DMEM containing 5.5-mM glucose, 10% FBS, 100-µM BrdU, 100 U/ml of penicillin, and 100 mg/ml of streptomycin in a humidified atmosphere of 5% CO_2_ at 37 °C. NRVMs were confirmed by morphological examination. NRVMs were then placed in a serum-free medium overnight before the experiments.

For high glucose (HG)-induced cardiomyocyte apoptosis, NRVMs were exposed to D-glucose at a final concentration of 45 mM for 72 h and exposed to 5.5-mM glucose as control. The identical concentration of mannitol was added to control to exclude a hyperosmolar effect. In the reactive oxygen species (ROS)-related experiment, 5-mM N-acetylcysteine (NAC, ST1546, Beyotime, Shanghai) was used to inhibit ROS at the time of HG stimulation [52]. The 50 μmol/L of H_2_O_2_ as a positive control could induce the production of ROS and upregulation of TRPM2 level in NRVMs within 72 h. The medium was replaced every 12 h to maintain the H_2_O_2_ concentration [52]. The cell treatments were described in the corresponding figure legends.

#### ShRNA preparation and infection of primary cardiomyocytes

The shRNA of TRPM2 was constructed and synthesized by GeneChem (Shanghai, China). A shRNA sequence targeting rat *TRPM2* gene (GenBank NM_294329) (5′-ccggTTGGAACAAGCTATGCTTGATctcgagATCAAGCATAGCTTGTTCCAAtttttg-3′) and a scrambled (5′-ccggTTCTCCGAACGTGTCACGTctcgagACGTGACACGTTCGGAGAAtttttg-3′) sequence were cloned into the U6-MCS-Ubiquitin-Cherry-IRES-puromycin lentiviral vector (Addgene plasmid GV298). Lentiviruses were generated by transiently transfecting the resulting lentiviral vectors along with packaging vectors pMDLg/pRRE, pRSV-Rev, and pMD2.G into HEK 293 T cells (from the human embryonic kidney cell line) using Lipofectamine 2000. The medium containing viruses was harvested 48 h following the start of transfection and concentrated using Millipore Centricon Plus-20 filters. For lentivirus infection, viral solutions (MOI = 50) were added to cell culture medium containing polybrene (5 μg/ml, H8761, Solarbio, Beijing). After 8 h of infection, fresh DMEM medium was replaced. 48 h after infection, NRVMs were selected using puromycin (1.5 μg/ml, P8230, Solarbio, China). The efficiency of shRNA transfection was determined with western blot analysis. The transfected cells were then treated with HG for the subsequent studies.

#### In vitroROS production measurement

ROS was detected using a 2′,7′-Dichlorofluorescin diacetate (DCF-DA) reagent (35,845, Sigma, USA). NRVMs were seeded in six-well plates and treated with different reagents for 72 h. Then, medium was removed and replaced with the serum-free medium containing 10-µM DCF-DA for 30 min at 37 °C. Subsequently, the cells were washed with PBS for three times and then fluorescent intensity was measured by the flow cytometer using wavelengths of 488/525 nm for excitation/emission, respectively.

#### MEK1/2 inhibitor and mTOR agonist

The MEK1/2 inhibitor U0126 (A1337) and mTORC1 agonist MHY1485 (B5853) were purchased from APExBIO (Shanghai, China). NRVMs were treated with 20-µM U0126 or MHY1485 for one hour to inhibit the MEK1/2 pathway or activate the mTORC1 pathway before exposure to 45 mM of HG stimulation for 72 h.

#### Cell viability detection

Viability was determined by a Cell Counting Kit-8 (CCK-8, C0037, Beyotime, China) according to the manufacturer’s protocol. Briefly, NRVMs were seeded onto 96-well plates (1000 cell per well) and subsequently treated with HG as indicated. Subsequently, cells were incubated with 10-µmol CCK-8 solutions under normal incubation conditions for 2 h. The absorbance at 450 nm, as an indicator of cell viability, was measured using a microplate reader (Bio-Rad, USA). Cell viability was calculated based on the relative optical density compared with that of untreated controls.

#### Cell apoptosis assay

Cell apoptosis was assayed using the fluorescein isothiocyanate (FITC) Annexin V Apoptosis Detection Kit (KGA108, KeyGEN BioTECH, China) following the manufacturer’s instructions. In brief, NRVMs were washed in PBS three times and resuspended in 400 µL of binding buffer with FITC annexin V and Propidium Iodide (PI, 5 µL each). The cell suspension was incubated for 15 min at room temperature in the dark and analyzed by flow cytometry (FACSCanto II; BD, San Jose, CA, USA) within 1 h. The index of apoptosis was expressed as the percentage of total apoptotic cells, which included the percentage of early apoptotic cells (Annexin V positive and PI negative) plus the percentage of late apoptotic cells (Annexin V positive and PI positive).

#### Real-time quantitative PCR (RT-qPCR)

Total RNA was extracted from the kidney tissues using Trizol reagents (Invitrogen Life Technologies, Carlsbad, CA) according to the manufacturer’s protocol. The cDNA synthesis was performed using a TaqMan reverse transcription kit (#KR118-03, Tiangen, Beijing, China). Then, quantitative real-time PCR was performed using a SuperReal PreMix plus (SYBR Green) Kit (#FP205, Tiangen, China) on a 7500 Fast Real-Time PCR system from Applied Biosystems (Bio-Rad, USA). All RT-PCR analysis was performed in duplicate 20-μL reactions in 96-well plates. The resulting melting curves were visually inspected to ensure specificity of product detection. The relative gene expression levels were determined by the 2^–ΔΔCT^ using GAPDH as a reference gene. All primer sequences are listed in *Supplementary Table 1*.

#### Western blot analysis

Kidney tissues or HK2 cells were homogenized and lysed using radioimmunoprecipitation (RIPA) buffer (#R0010, Solarbio, Beijing) supplemented with cocktail proteinase and phosphatase inhibitors (#P6730 and #P1260, Solarbio, Beijing, China). Protein concentration was measured with the BCA Protein Assay Kit (#PA115, Tiangen, Beijing). Equal amounts of protein were separated on SDS-PAGE electrophoresis and electrotransferred onto PVDF membranes (Millipore, USA). The membranes were blocked in TBST buffer and then further incubated primary antibodies (Supplementary Table 2) overnight at − 4 °C. Then, immunoreactive bands were detected through incubating with secondary antibody (Boster, Wuhan, China) conjugated with horseradish peroxidase (HRP) and visualized using enhanced chemiluminescence reagents (ECL, Thermo Fisher Scientific).

#### Statistical analysis

Data were presented as mean ± standard error (SE) using the GraphPad Prism 8.0 software (GraphPad Software Inc., CA, USA). Differences were evaluated using unpaired Student’s t test between two groups. One-way ANOVA was conducted followed by LSD post hoc test for comparisons between multiple groups. The non-normal distributed data were analyzed using non-parametric testing (Mann–Whitney *U* test for two groups and Kruskal–Wallis H test for multiple groups). Statistical analysis was performed using SPSS 23.0 (SPSS Inc., Chicago, IL, USA) software and *P* < 0.05 was considered statistically significant.

## Results

### Dynamic change of FBG and body weight in each group

Compared to the control arms, the mice with HFD/STZ-induced diabetes exhibited hyperglycemia peaking nearly 20 mmol/L indicated metabolic disturbance (*Supplementary Table 3*). However, TRPM2 knockdown did not change FBG level in the diabetic mice, suggesting that TRPM2 did not affect DCM through regulating glucose metabolism. In addition, HFD/STZ-induced diabetic mice showed no significant weight loss considering the visceral obesity caused by HFD. Meanwhile, TRPM2 knockdown did not change body weight level in the diabetic mice (Supplementary Table 4).

### Inflammatory cell infiltration

**Fig. 1:**
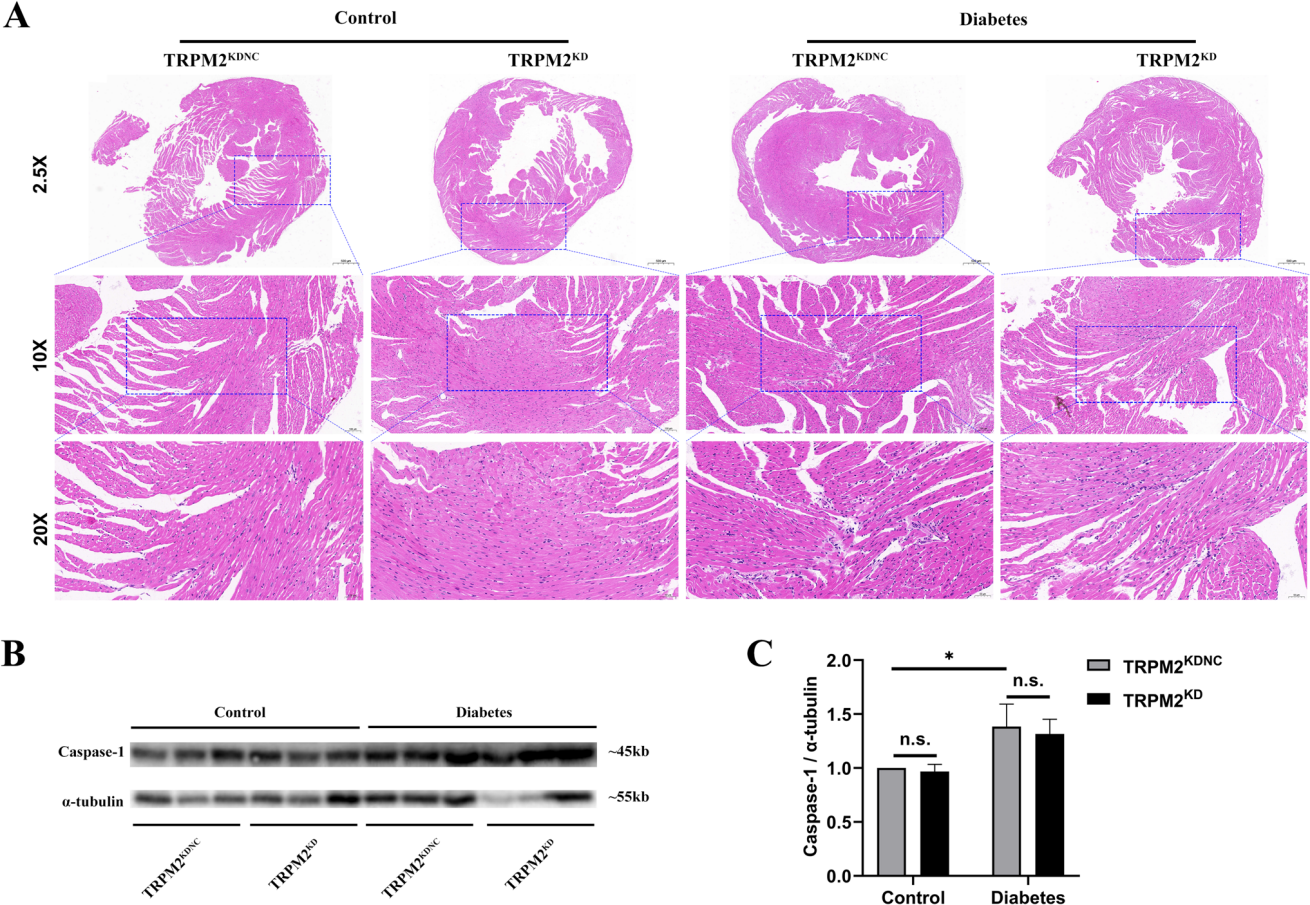
Inflammatory cell infiltration in the myocardium. **A** Representative images of inflammatory cell infiltration into the myocardium according to H&E staining from the different groups (*n* = 5 per group, magnification = 200×). The myocardial interstitium was mainly infiltrated by mononuclear macrophages. **B** Representative western blot image of Caspase-1 in the myocardium from the different groups. α-tubulin was used as a loading control. **C** Corresponding densitometric analysis of blots in B (*n* = 6 per group). The data are represented as the means ± SE; *P < 0.05

Compared to the control groups, the mice with HFD/STZ-induced diabetes exhibited inflammatory cell infiltration into the myocardium according to H&E staining (Fig. 1A). The myocardial interstitium was mainly infiltrated by mononuclear macrophages. However, TRPM2 knockdown did not improve inflammatory infiltration in the diabetic hearts. Besides, western blotting showed that compared to the control arms, the protein expression of Caspase-1 in the myocardium of HFD/STZ-induced diabetic mice was increased, while TRPM2 knockdown did also not restore the protein expression of Caspase-1 in the diabetic hearts (Fig. 1B, C). These findings suggested that TRPM2 knockdown did not ameliorate DCM by the regulation of myocardial inflammation.

### Cardiac fibrosis

Compared to the control groups, the mice with HFD/STZ-induced diabetes exhibited significant collagen matrix deposition in the myocardium according to Masson’s trichrome staining. However, TRPM2 knockdown did not attenuate HFD/STZ-induced myocardial fibrosis in the diabetic hearts (Supplementary Fig. 1A, B). Moreover, HFD/STZ-induced diabetic mice displayed a significant increasement of α-SMA-positive cells as shown by immunohistochemistry (Supplementary Fig. 1C, D), which accompanied by the increased protein expression of collagen type I in the myocardium (Supplementary Fig. 1E, F). However, TRPM2 knockdown did not reduce the expression of α-SMA-positive cells and collagen type I in the diabetic hearts. These findings suggested that TRPM2 knockdown did not ameliorate cardiac fibrosis in DCM.

### The mRNA expression of myocardial hypertrophy or fibrosis markers

Compared to the control groups, HFD/STZ-induced diabetic mice did not displayed increased cardiac fetal gene reactivation, such as atrial natriuretic peptide (ANP) and brain natriuretic peptide (BNP) detected by RT-qPCR in the myocardium (Supplementary Fig. 2A, B). TRPM2 knockdown did also not change the mRNA expression of ANP and BNP. Furthermore, compared to the control arms, HFD/STZ-induced diabetic mice exhibited increased mRNA expression of collagen type I (*P* = 0.082) and III (*P* = 0.028) in the myocardium (Supplementary Fig. 2C,D). Nonetheless, TRPM2 knockdown did not reduce the mRNA expression of collagen type I and III.

### The expression of TRPM2

**Fig. 2:**
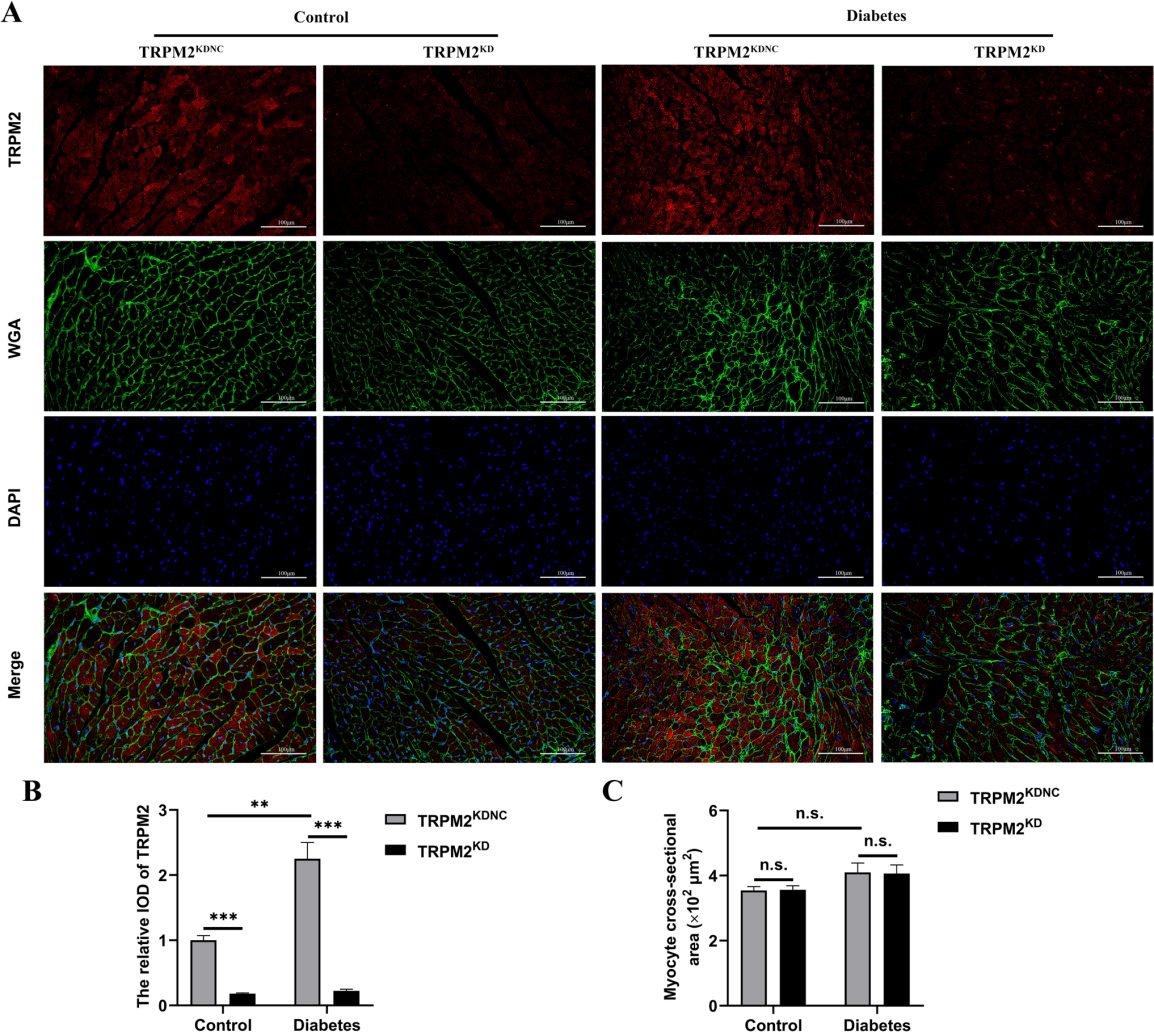
Immunofluorescent double staining of TRPM2 and WGA in the myocardium. **A** Representative images of IF double staining of TRPM2 and WGA in mice hearts from the different groups. **B** Corresponding statistic analysis of TRPM2 fluorescence intensity in A (*n* = 5 per group). **C** Corresponding statistic analysis of cardiomyocyte size used WGA staining in A (*n* = 5 per group). The data are represented as the means ± SE; **P < 0.01 and ***P < 0.001

**Fig. 3:**
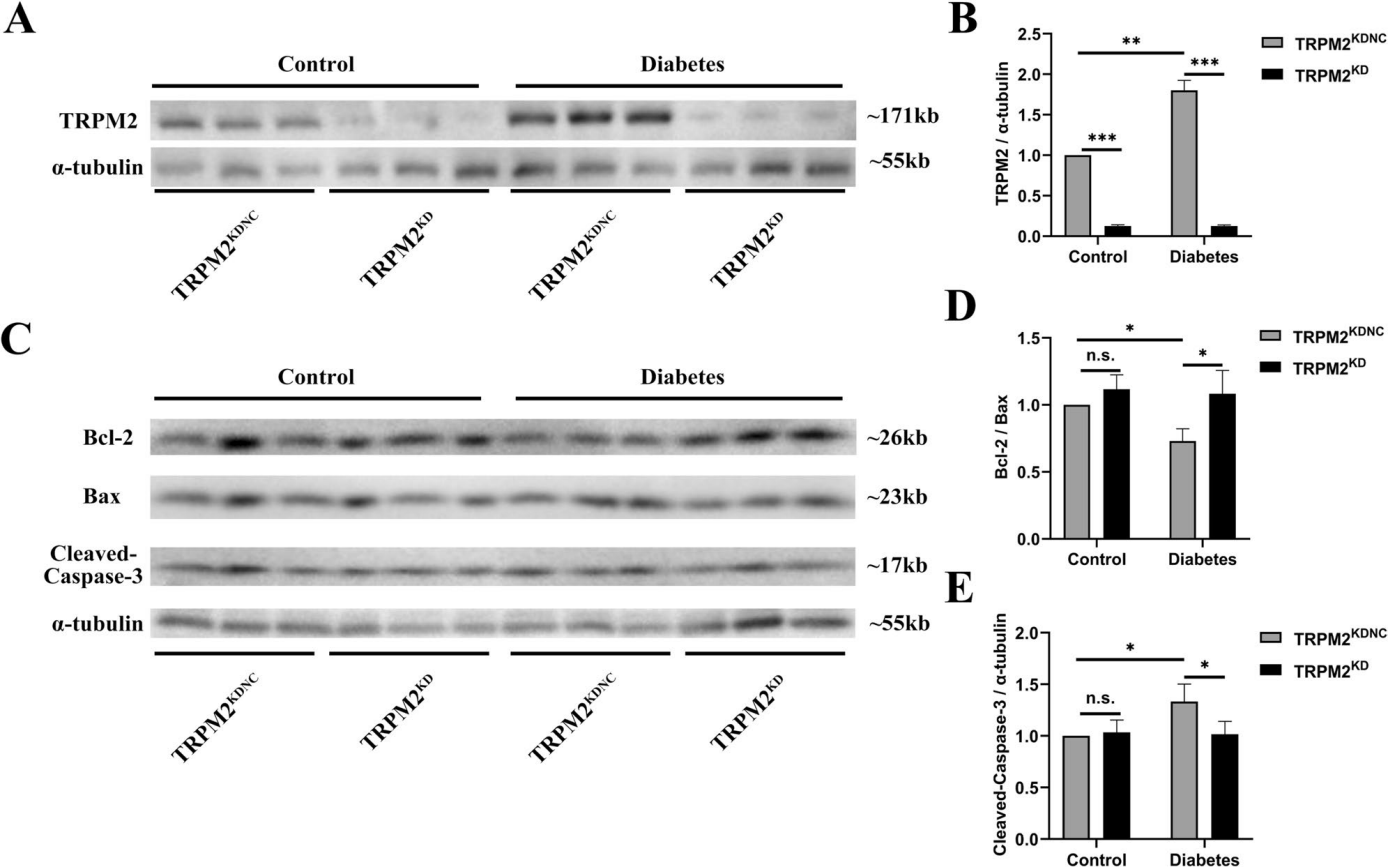
The expression of TRPM2 and apoptosis-related proteins. **A** Representative western blot image of TRPM2 in the myocardium from the different groups. α-tubulin was used as a loading control. **B** Corresponding densitometric analysis of blots in B (*n* = 6 per group). **C** Representative western blot image of Bax, Bcl-2, and cleaved caspase-3 in the myocardium from the different groups. α-tubulin was used as a loading control. (F) Corresponding densitometric analysis of blots in C (*n* = 6 per group). The data are represented as the means ± SE; *P < 0.05, **P < 0.01, and ***P < 0.001

Compared to the control group, HFD/STZ-induced diabetic mice exhibited enhanced fluorescence intensity of TRPM2 (Fig. 2A, B). To explore whether TRPM2 knockdown alleviated DCM injury under diabetic conditions, we knocked down TRPM2 expression with by a single caudal vein injection of ~ 1 × 10^11^ genome copies of recombinant AAV9-U6-shTRPM2 at 6 weeks of age in the corresponding mice. Immunofluorescent double staining indicated the TRPM2 fluorescence intensity was decreased in the myocardium from the hearts of AAV9-U6-shTRPM2-injected mice (Fig. 2A, B). Besides, compared to the control group, HFD/STZ-induced diabetic mice exhibited significant increased protein level of TRPM2 (Fig. 3A, B). Protein quantification confirmed that the protein level of TRPM2 was effectively knocked down in the hearts of AAV9-U6-shTRPM2-injected mice (Fig. 3A, B).

In addition, this study used WGA staining to quantify in vivo cardiomyocyte size and evaluate cardiac hypertrophy, and the results showed that HFD/STZ-induced diabetic mice did not displayed significant larger cardiomyocyte size compared to the control arms (Fig. 2A, C). TRPM2 knockdown did also not change the cardiomyocyte size.

### TRPM2 knockdown inhibited myocardial apoptosis in HFD/STZ-induced diabetic mice

Hyperglycemia-induced myocardial apoptosis is partly mediated by activation of the cytochrome c-activated caspase-3 pathway. Western blotting showed that compared to the control arms, the protein expression of cleaved caspase-3 and pro-apoptotic Bax increased obviously, of antiapoptotic Bcl-2 decreased significantly in the myocardium of HFD/STZ-induced diabetic mice (Fig. 3C–E). However, TRPM2 knockdown ameliorated the expression changes of apoptosis-related proteins in the myocardium of HFD/STZ-induced diabetic mice.

**Fig. 4:**
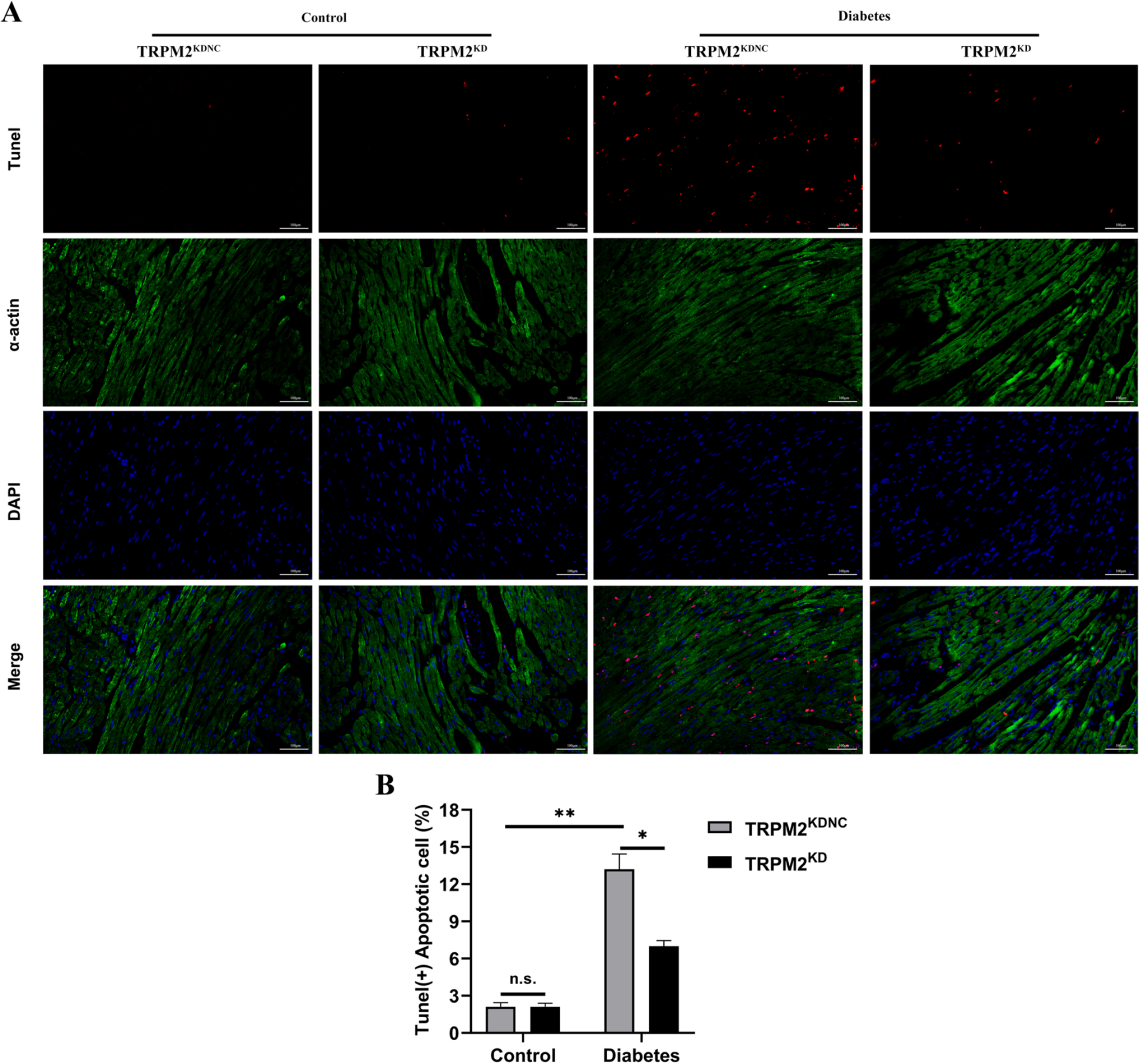
Myocardial apoptosis detected by the TUNEL staining. **A** Representative images of TUNEL staining in the myocardium from the different groups. α-actin staining was used for co-localization of cardiomyocytes and DAPI was used for nuclear staining. **B** Corresponding statistic analysis of cardiomyocyte apoptosis in A (*n* = 5 per group). The data are represented as the means ± SE; *P < 0.05 and **P < 0.01

To determine the effects of TRPM2 knockdown on HFD/STZ-induced myocardial apoptosis, we performed TUNEL staining to assess cardiomyocyte apoptosis. HFD/STZ-induced diabetic mice showed a higher proportion of apoptosis in cardiac myocytes as compared to that in controls, which was reversed by TRPM2 knockdown (Fig. 4A, B). These findings suggested that TRPM2 knockdown inhibited cardiomyocyte apoptosis in HFD/STZ-induced diabetic mice.

### TRPM2 knockdown promoted myocardial-lessened autophagy in HFD/STZ-induced diabetic mice

**Fig. 5:**
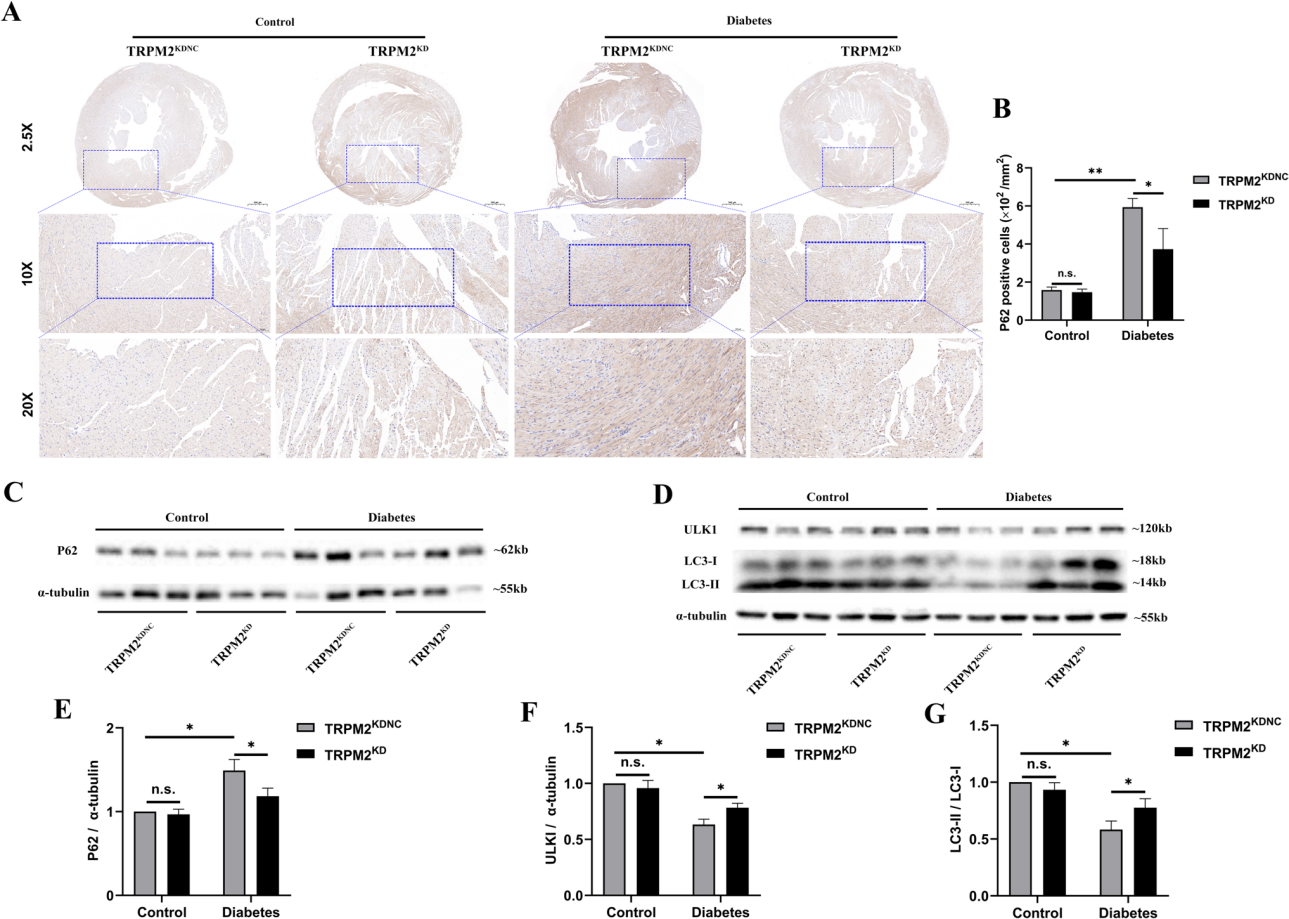
TRPM2 knockdown inhibited myocardial excessive autophagy in HFD/STZ-induced diabetic mice. **A** Immunohistochemical analysis of autophagy substrates P62 protein in mice hearts from the different groups (magnification = 200×). **B** Corresponding statistic analysis of P62 in A (*n* = 5 per group). **C** Representative western blot image of P62 in the myocardium in each group. α-tubulin was used as a loading control. **D** Heart homogenates were analyzed by western blot using an antibody against LC3 II/I and ULK1 proteins. α-tubulin was used as a loading control. **E** Corresponding densitometric analysis of blots in C (*n* = 6 per group). **F**, **G** Corresponding densitometric analysis of blots in D (*n* = 6 per group). The data are represented as the means ± SE; *P < 0.05 and **P < 0.01

To determine the effects of TRPM2 knockdown on HFD/STZ-induced myocardial-lessened autophagy, we performed immunohistochemical analysis of P62 protein (an indicator of autophagic flux) and western blotting of autophagy-related proteins to assess cardiomyocyte autophagy. HFD/STZ-induced diabetic mice showed a higher proportion of P62-positive cells as shown by immunohistochemistry as compared to that in controls, which was ameliorated by TRPM2 knockdown (Fig. 5A, B).

Western blotting showed that compared to the control arms, the protein expression of P62 increased significantly of ULK1 and LC3-II/LC3-I decreased obviously in the myocardium of HFD/STZ-induced diabetic mice, which was also reversed by TRPM2 knockdown (Fig. 5C–G). Next, the ultrastructural morphologies of the hearts were observed by transmission electron microscopy. HFD/STZ-induced diabetic mice showed a lower proportion of autophagic-like vesicles as compared to that in controls, which was reversed by TRPM2 knockdown (Supplementary Fig. 3A, B). These findings suggested that TRPM2 knockdown promoted myocardial-lessened autophagy in HFD/STZ-induced diabetic mice.

### TRPM2 knockdown improved systolic and diastolic function

**Fig. 6:**
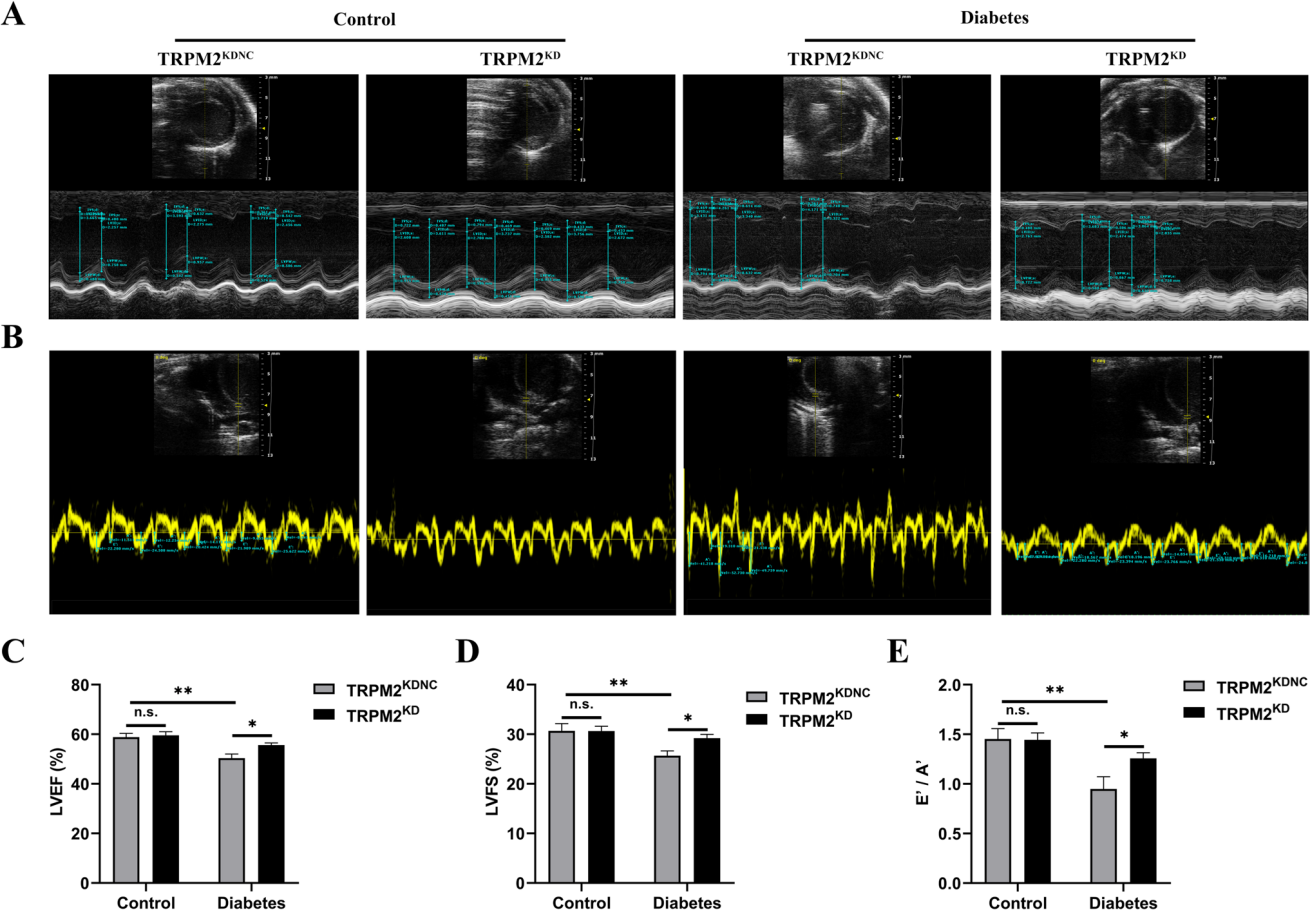
Cardiac function in mice measured by echocardiography. **A** Representative images by M-mode echocardiography in mice hearts from the different groups. **B** Representative images by pulsed wave Doppler echocardiography in mice hearts from the different groups. **C**–**E** Echocardiography analysis showing cardiac systolic dysfunction assessed by LVEF (**C**) and LVFS (**D**) diastolic dysfunction assessed by ratio of diastolic mitral annulus velocities (E′/A′) (**E**) (*n* = 5 per group). The data are represented as the means ± SE; *P < 0.05 and **P < 0.01

Cardiac analysis by echocardiography demonstrated HFD/STZ-induced diabetic mice showed reduced LVEF and LVFS indicated worse cardiac systolic dysfunction as compared to that in controls, which was ameliorated by TRPM2 knockdown (Fig. 6A, C, D). Because the mice were older at the time of sacrifice (32 week old), the LVEF was only 60% in control group. Moreover, significant increases in E′/A′ ratio were observed in HFD/STZ-induced diabetic mice that indicated worse cardiac diastolic dysfunction, which was reversed by TRPM2 knockdown (Fig. 6B,E).

### Changes in MEK/ERK signaling pathway

**Fig. 7:**
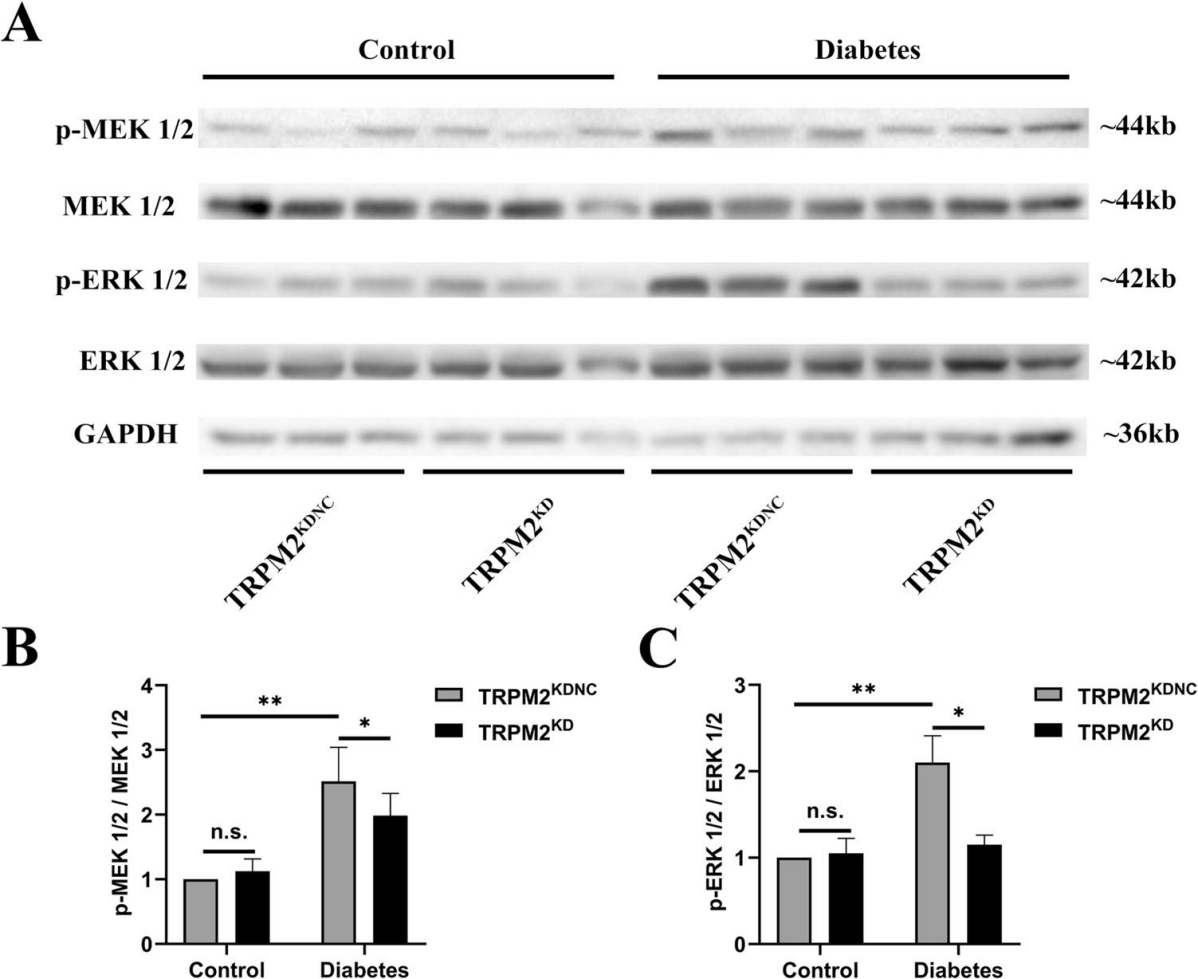
Changes in MEK/ERK signaling pathway. **A** Representative western blot analyses of p-MEK1/2 (Ser217/221) and MEK1/2, p-ERK1/2 (Thr202/Tyr204) and ERK1/2 in mice heart homogenates from the different groups. GAPDH was used as a loading control. **B** Corresponding densitometric analysis of blots in A (*n* = 6 per group). The data are represented as the means ± SE; *P < 0.05 and **P < 0.01

HFD/STZ-induced diabetic mice exhibited increased protein expression levels of p-MEK1/2 (Ser217/221) and p-ERK1/2 (Thr202/Tyr204) in heart homogenates as compared to that in controls, which was reversed by TRPM2 knockdown (Fig. 7 A/B).

#### Changes in mTORC1 signaling pathway

**Fig. 8:**
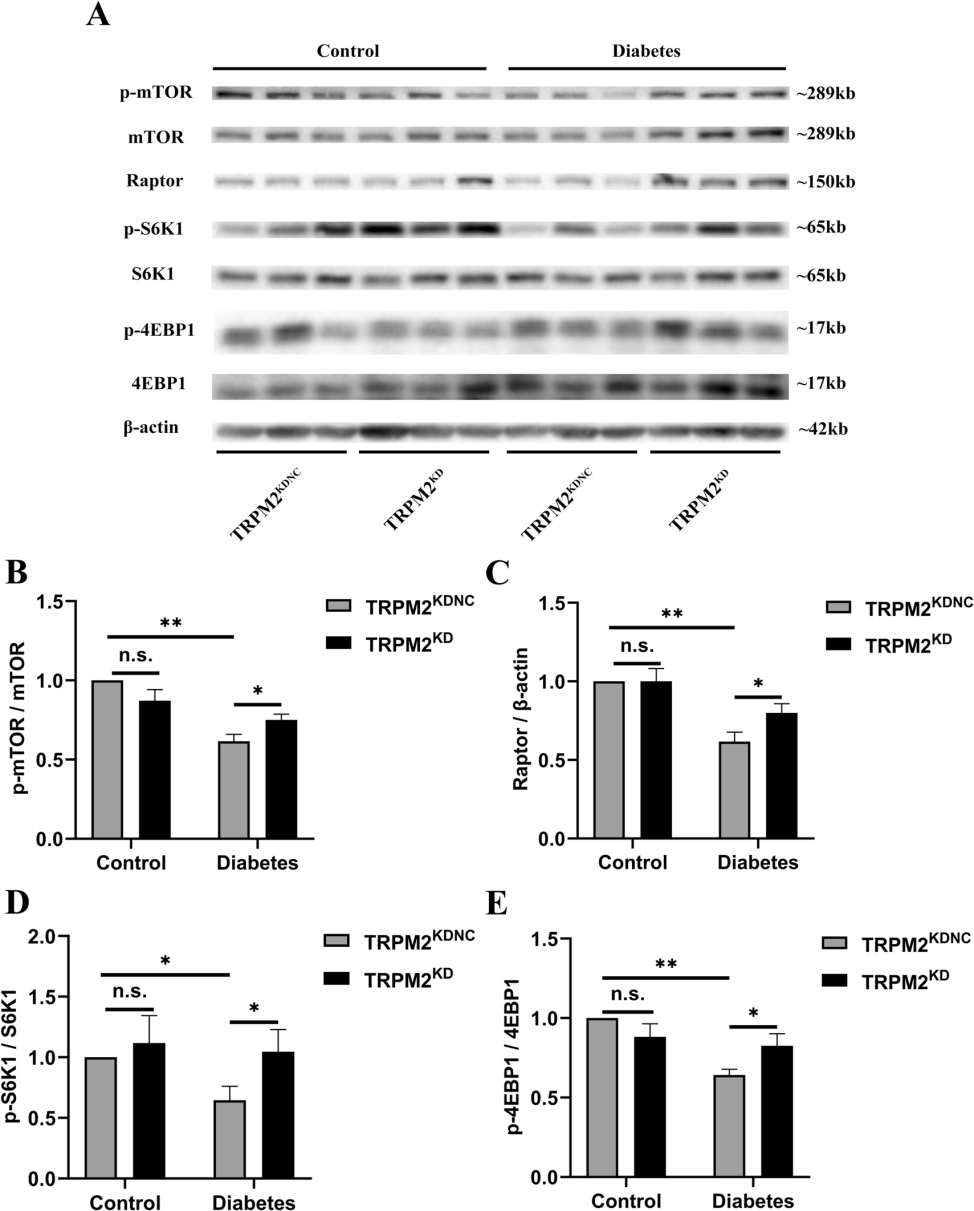
Changes in mTORC1 signaling pathway. **A** Representative western blot analyses of p-mTOR (Ser2448) and mTOR, Raptor, p-S6K1 (Thr389) and S6K1, and p-4EBP1 (Thr37/46) and 4EBP1 in mice heart homogenates from the different groups. β-actin was used as a loading control. **B** Corresponding densitometric analysis of blots in A (*n* = 6 per group). The data are represented as the means ± SE; *P < 0.05 and **P < 0.01

HFD/STZ-induced diabetic mice exhibited an down-regulated protein expression levels of p-mTOR (Ser2448), Raptor, p-S6K1 (Thr389), and p-4EBP1 (Thr37/46) in heart homogenates as compared to that in controls, which was also reversed by TRPM2 knockdown (Fig. 8).

#### High glucose-induced oxidative stress increased TRPM2 expression

**Fig. 9:**
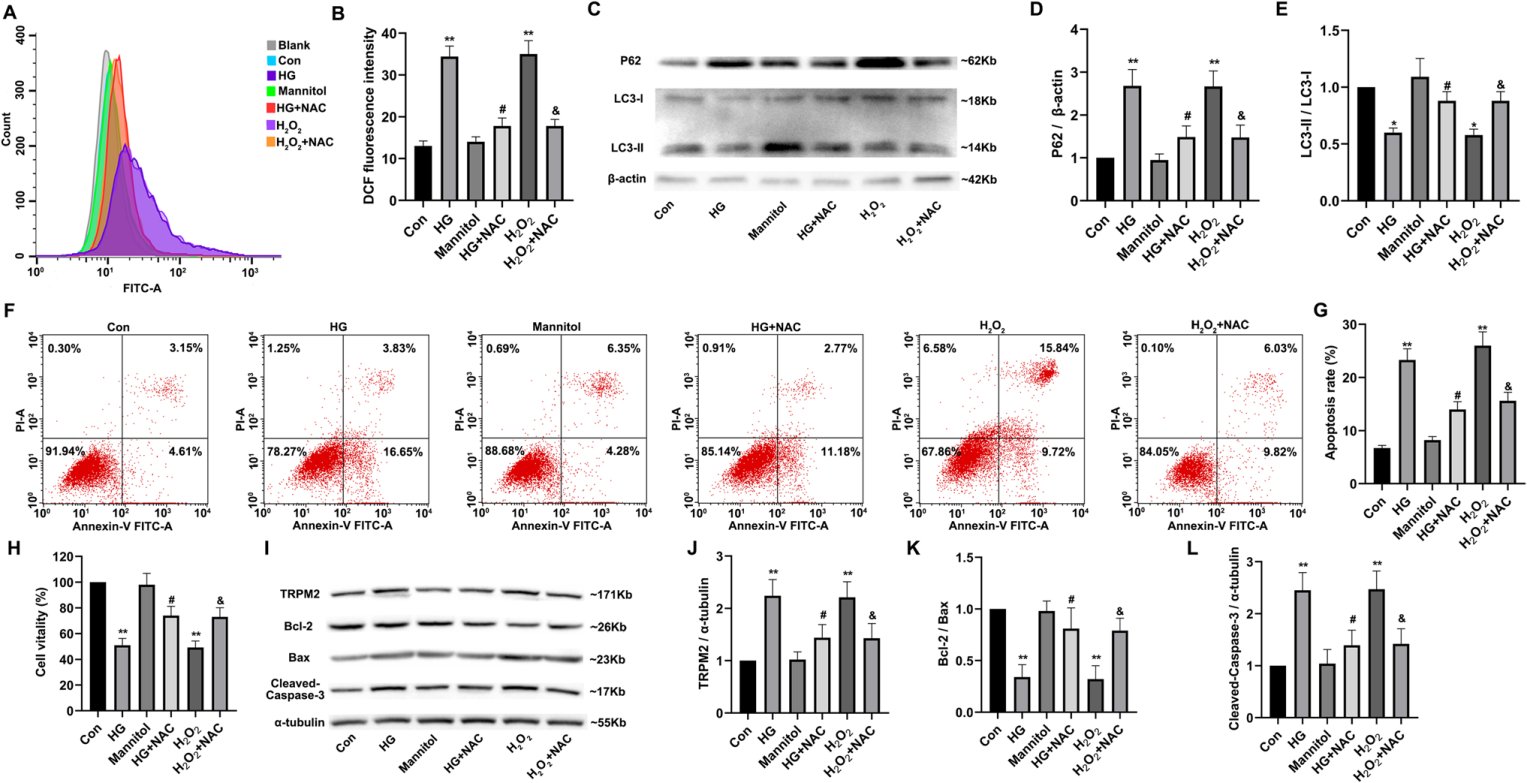
High glucose-induced oxidative stress increased TRPM2 expression. Primary cardiomyocytes were treated with 45-mM high glucose (HG) for 72 h to simulate diabetic myocardial injury in vivo. In addition, 5-mmol/L N-acetylcysteine (NAC) was used to inhibit ROS produced by primary cardiomyocytes stimulated by HG, and 50-μmol/L H_2_O_2_ treatment was regarded as a positive control group inducing ROS production in primary cardiomyocytes. The medium was changed every 12 h to maintain H_2_O_2_ concentration. **A**, **B** Intracellular ROS production in primary cardiomyocytes was detected with DCF-DA reagent and corresponding statistic analysis. Compared to that in controls, ROS levels significantly increased after HG stimulation for 72 h in primary cardiomyocytes. There was no significant change in the high osmotic pressure group (mannitol treatment) compared to control group, but the ROS level in the HG group was decreased by pre-incubation with NAC. H_2_O_2_-positive control group could also increase the ROS level of cardiomyocytes, while NAC pre-incubation decreased ROS levels in H_2_O_2_-positive control group. **C** Representative western blots of P62 and LC3 protein in primary cardiomyocytes from the different groups. β-actin was used as a loading control. **D**, **E** Corresponding densitometric analysis of blots in C. Compared to that in controls, the protein expression level of autophagy substrate P62 was significantly increased after stimulated with HG for 72 h, the ratio of LC3-II/LC3-I was also significantly decreased, and there was no significant change in the high osmotic pressure group, suggesting that HG inhibited autophagy in primary cardiomyocytes. However, NAC pre-incubation could partially recover the changes of the protein expression level of autophagy-related proteins in HG group. H_2_O_2_-positive control group could also increase the protein expression level of P62, reduced the ratio of LC3-II/LC3-I, while pre-NAC incubation could partially recover the changes of autophagy-related proteins in H_2_O_2_-positive control group. **F**, **G** The apoptosis in different treatment groups was detected by flow cytometry and corresponding statistic analysis. **H** The cell viability was measured by cell counting kit-8. Compared to that in controls, the cell viability of primary cardiomyocytes decreased significantly after stimulated with HG for 72 h, and the apoptosis level was also significantly increased, while NAC pre-incubation could partially restore the cell viability and resist apoptosis in the HG group. **I** Representative western blots of TRPM2, Bcl-2, Bax, and cleaved Caspase-3 protein in primary cardiomyocytes from the different groups. α-tubulin was used as a loading control. **J**–**L** Corresponding densitometric analysis of blots in I. Compared to that in controls, the protein expression level of TRPM2 and cleaved Caspase-3 significantly increased and the ratio of Bcl-2/Bax decreased significantly, in primary cardiomyocytes after stimulated with HG for 72 h. However, NAC pre-incubation could partially recover the changes of the protein expression level of TRPM2 and apoptotic proteins in HG group. The cell experiment was repeated independently for three times. The data are represented as the means ± SE; *P < 0.05, **P < 0.01 versus control group, ^#^*P* < 0.05 versus HG group, ^#&^*P* < 0.05 versus H_2_O_2_ group

As shown in Fig. 9A,B, compared to that in controls, ROS levels significantly increased after HG stimulation for 72 h in primary cardiomyocytes, but the ROS level in the HG group was decreased by pre-incubation with NAC. As shown in Fig. 9C–E, compared to that in controls, the protein expression level of autophagy substrate P62 significantly increased, and the ratio of LC3-II/LC3-I was also significantly decreased after HG stimulation, suggesting that HG inhibited autophagy in primary cardiomyocytes. However, NAC pre-incubation could partially recover the changes of the protein expression level of autophagy-related proteins in HG group.

Compared to that in controls, the cell viability of primary cardiomyocytes decreased significantly, and the apoptosis level was also significantly increased after HG stimulation, while NAC pre-incubation could partially restore the cell viability and resist apoptosis in the HG group (Fig. 9F–H). Compared to that in controls, the protein expression level of TRPM2 and cleaved Caspase-3 significantly increased, the ratio of Bcl-2/Bax decreased, in primary cardiomyocytes after HG stimulation. However, NAC pre-incubation could partially recover the changes of the protein expression level of TRPM2 and apoptotic proteins in HG group (Fig. 9I–L). These results suggested that after high-glucose stimulation of primary cardiomyocytes, the induced oxidative stress could increase the expression level of TRPM2 protein and trigger the apoptosis and autophagy inhibition.

#### TRPM2 silencing defended against high glucose-induced apoptosis and autophagy inhibition in primary cardiomyocytes

**Fig. 10:**
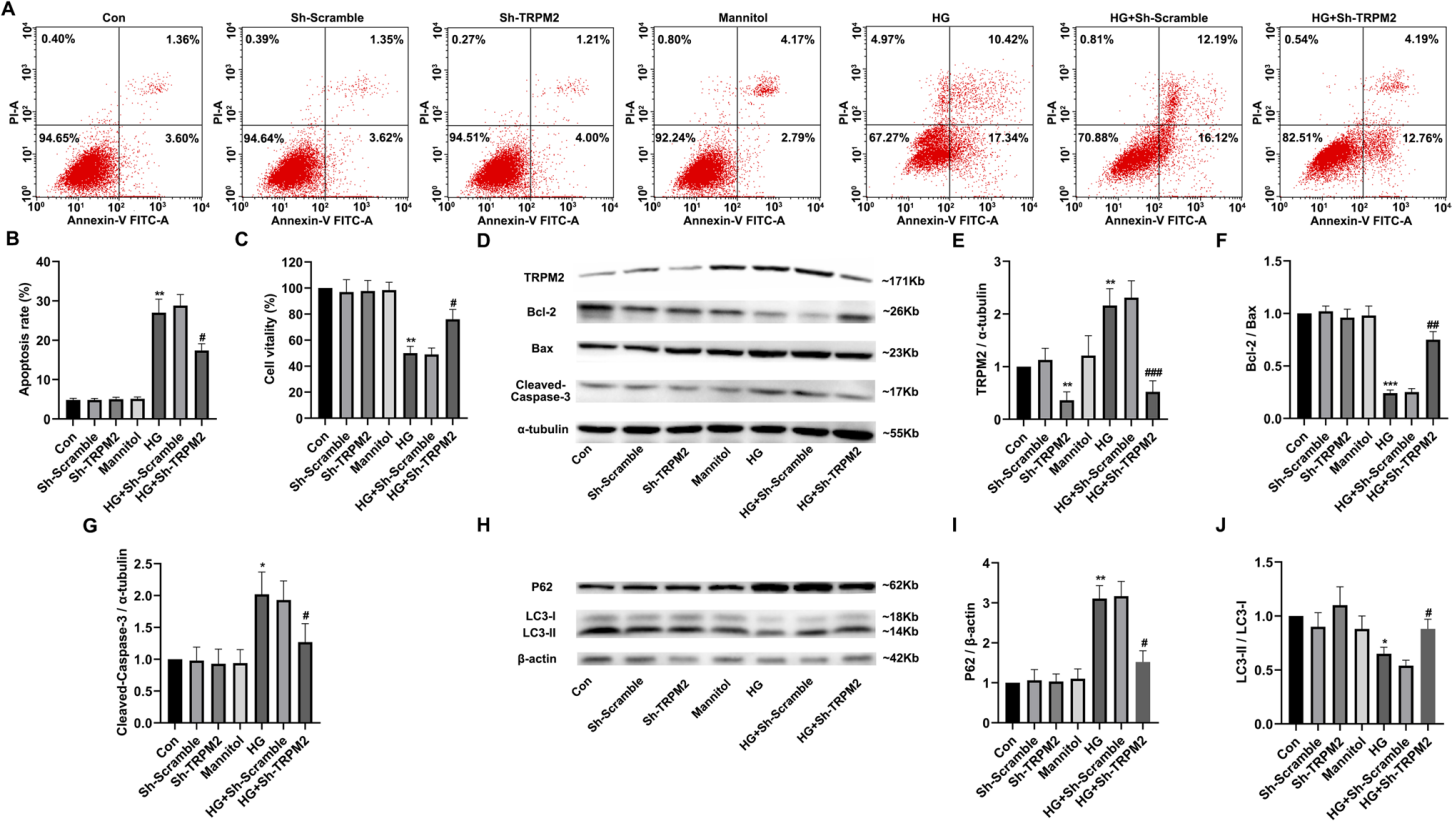
TRPM2 silencing defended against high glucose-induced apoptosis and autophagy inhibition in primary cardiomyocytes. Primary cardiomyocytes were infected with lentivirus for 72 h and then treated with normal glucose or 45-mM high glucose (HG) for 72 h. **A**, **B** The apoptosis in different treatment groups was detected by flow cytometry and corresponding statistic analysis. Compared to that in controls, the apoptosis level of primary cardiomyocytes was significantly increased after 45-mM HG stimulation for 72 h, and there was no significant change in the hyperosmotic pressure group. However, *TRPM2* silencing by lentivirus infection of primary cardiomyocytes could resist the apoptosis of HG group. **C** The cell viability was measured by cell counting kit-8. Compared to that in controls, the cell viability of primary cardiomyocytes decreased significantly after stimulated with HG for 72 h, and there was no significant change in the hyperosmotic pressure group. However, the vitality of primary cardiomyocytes in HG group could be partially restored after *TRPM2* silencing. **D** Representative western blots of TRPM2, Bcl-2, Bax, and cleaved Caspase-3 protein in primary cardiomyocytes from the different groups. α-tubulin was used as a loading control. **J**–**L** Corresponding densitometric analysis of blots in D. Compared to that in controls, the protein expression level of TRPM2 and cleaved Caspase-3 significantly increased, the ratio of Bcl-2/Bax decreased, in primary cardiomyocytes after 45-mM HG stimulation for 72 h, while there was no significant change in the high osmotic pressure group. However, the apoptotic protein changes in HG group could be partially restored after *TRPM2* silencing. **H** Representative western blots of P62 and LC3 protein in primary cardiomyocytes from the different groups. β-actin was used as a loading control. **I**, **J** Corresponding densitometric analysis of blots in H. Compared to that in controls, the protein expression level of autophagy substrate P62 significantly increased, ratio of LC3-II/LC3-I also significantly decreased after 45-mM HG stimulation for 72 h, while the protein expression level of autophagy-related proteins in HG group could be partially restored after *TRPM2* silencing. The cell experiment was repeated independently for three times. The data are represented as the means ± SE; *P < 0.05, **P < 0.01, ***P < 0.001 versus control group and ^#^*P* < 0.05 versus HG group

Compared to that in controls, the apoptosis level of primary cardiomyocytes significantly increased after HG stimulation, which was partially reversed by *TRPM2* silencing (Fig. 10A, B). Compared to that in controls, the viability of cardiomyocytes significantly decreased after HG stimulation, which was partially restored by *TRPM2* silencing (Fig. 10C). Compared to that in controls, the protein expression level of *TRPM2* and cleaved Caspase-3 significantly increased and the ratio of Bcl-2/Bax significantly decreased, in primary cardiomyocytes after HG stimulation, which was partially restored by *TRPM2* silencing (Fig. 10D–G).

Compared to that in controls, the protein expression level of P62 significantly increased, and the ratio of LC3-II/LC3-I was also significantly decreased in primary cardiomyocytes after HG stimulation, which was partially reversed by *TRPM2* silencing (Fig. 10H–J). These results suggested that *TRPM2* silencing could partially recover the HG-induced apoptosis and autophagy inhibition in primary cardiomyocytes.

#### *TRPM2 silencing attenuated high glucose-induced apoptosis and autophagy inhibition in primary cardiomyocytes *via* regulating the MEK/ERK signaling pathway*

**Fig. 11:**
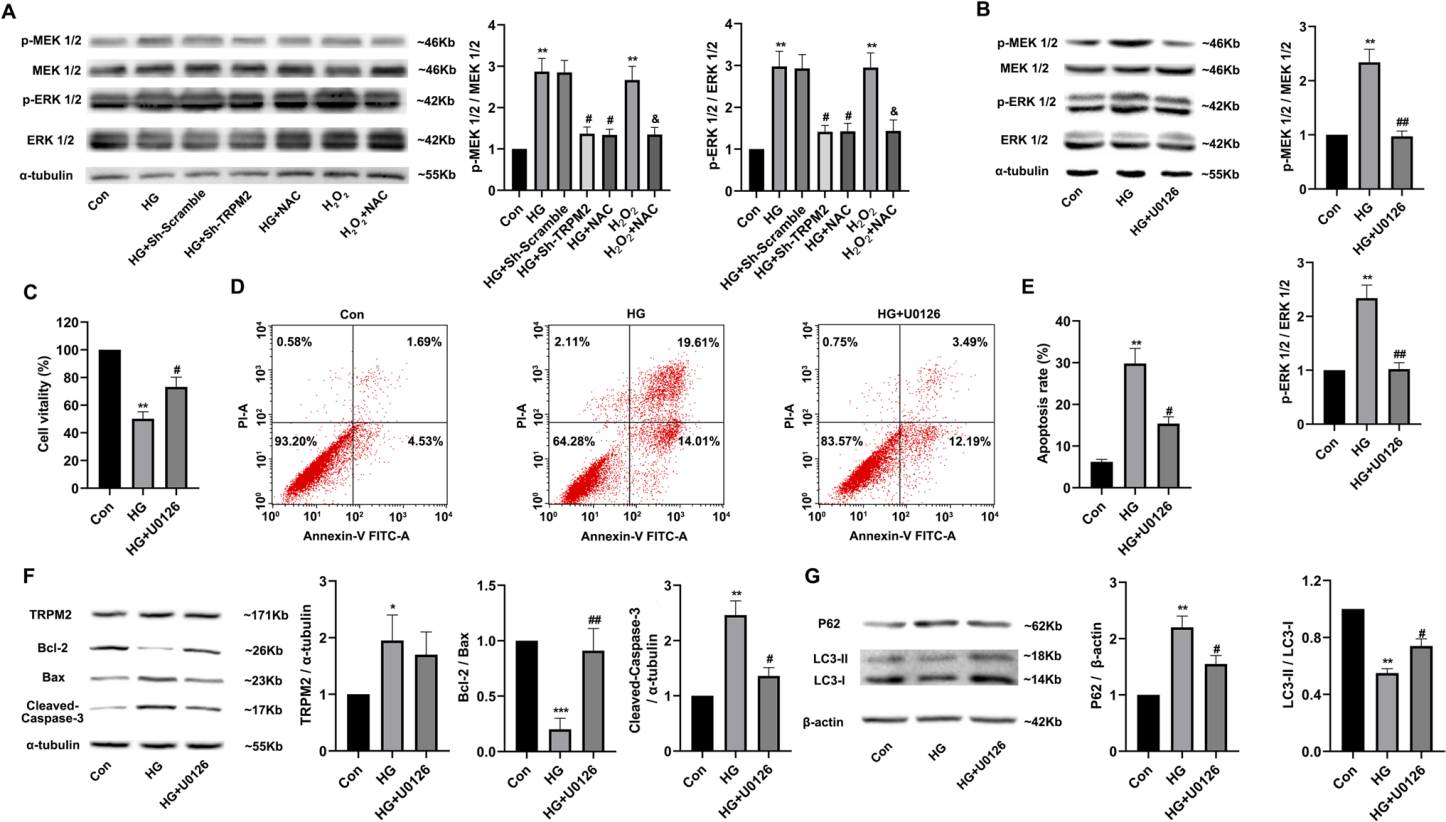
TRPM2 silencing attenuated high glucose-induced apoptosis and autophagy inhibition in cardiomyocytes via regulating the MEK/ERK pathway. **A** Representative blots of p-MEK1/2 (Ser217/221) and MEK1/2, p-ERK1/2 (Thr202/Tyr204), and ERK1/2 in high glucose (HG)-stimulated primary cardiomyocytes and corresponding densitometric analysis. α-tubulin was used as a loading control. Compared to that in controls, the protein expression levels of p-MEK1/2 and p-ERK1/2 were significantly increased after HG stimulation, which was reversed by *TRPM2* silencing. **B** Representative blots of MEK/ERK signaling pathway in HG-stimulated cardiomyocytes pre-incubated with U0126 and corresponding densitometric analysis. α-tubulin was used as a loading control. U0126 was widely used as an inhibitor of MEK/ERK signaling pathway. NRVMs were treated with 20-µM U0126 for one hour to inhibit the MEK1/2 pathway before exposure to 45 mM of HG stimulation for 72 h. The protein expression levels of p-MEK1/2 and p-ERK1/2 were significantly decreased by U0126 treatment in HG-stimulated cardiomyocytes after HG stimulation. **C** The cell viability was measured by cell counting kit-8 and corresponding statistic analysis. **D**, **E** The apoptosis was detected by flow cytometry and corresponding statistic analysis. Compared to that in controls, the cell viability decreased (**C**) and apoptosis increased **D**, **E** significantly in HG-stimulated cardiomyocytes, which was partially reversed by U0126 treatment. **F** Representative western blots of TRPM2, Bcl-2, Bax, and cleaved Caspase-3 protein in HG-stimulated cardiomyocytes pre-incubated with U0126 and corresponding densitometric analysis. α-tubulin was used as a loading control. Compared to that in controls, the protein expression level of cleaved Caspase-3 significantly increased; the ratio of Bcl-2/Bax significantly decreased, in primary cardiomyocytes after HG stimulation, which was partially restored by U0126 treatment. **G** Representative western blots of P62 and LC3 in HG-stimulated cardiomyocytes pre-incubated with U0126 and corresponding densitometric analysis. β-actin was used as a loading control. Compared to that in controls, the protein expression level of P62 significantly increased and ratio of LC3-II/LC3-I was also significantly decreased, in primary cardiomyocytes after HG stimulation, which was partially reversed by U0126 treatment. The cell experiment was repeated independently for three times. The data are represented as the means ± SE; *P < 0.05, **P < 0.01 versus control group and ^#^*P* < 0.05 versus HG group

Compared to that in controls, the protein expression levels of p-MEK1/2 (Ser217/221) and p-ERK1/2 (Thr202/Tyr204) were significantly increased after HG stimulation, which was reversed by *TRPM2* silencing (Fig. 11A). U0126 was widely used as an inhibitor of MEK/ERK signaling pathway (Fig. 11B). Compared to that in controls, the cell viability decreased (Fig. 11C) and apoptosis increased (Fig. 11D, E) significantly in cardiomyocytes after HG stimulation, which was also partially reversed by U0126 treatment. Compared to that in controls, the protein expression level of cleaved Caspase-3 significantly increased, and the ratio of Bcl-2/Bax significantly decreased in primary cardiomyocytes after HG stimulation, which was partially restored by U0126 treatment (Fig. 11F). Besides, compared to that in controls, the protein expression level of P62 significantly increased, and the ratio of LC3-II/LC3-I was also significantly decreased in primary cardiomyocytes after HG stimulation, which was partially reversed by U0126 treatment (Fig. 11G). These results suggested that *TRPM2* silencing might attenuate HG-induced apoptosis and autophagy inhibition in primary cardiomyocytes via regulating the MEK/ERK signaling pathway.

#### *TRPM2 silencing attenuated high glucose-induced apoptosis and autophagy inhibition in primary cardiomyocytes *via* regulating the mTORC1 signaling pathway*

**Fig. 12:**
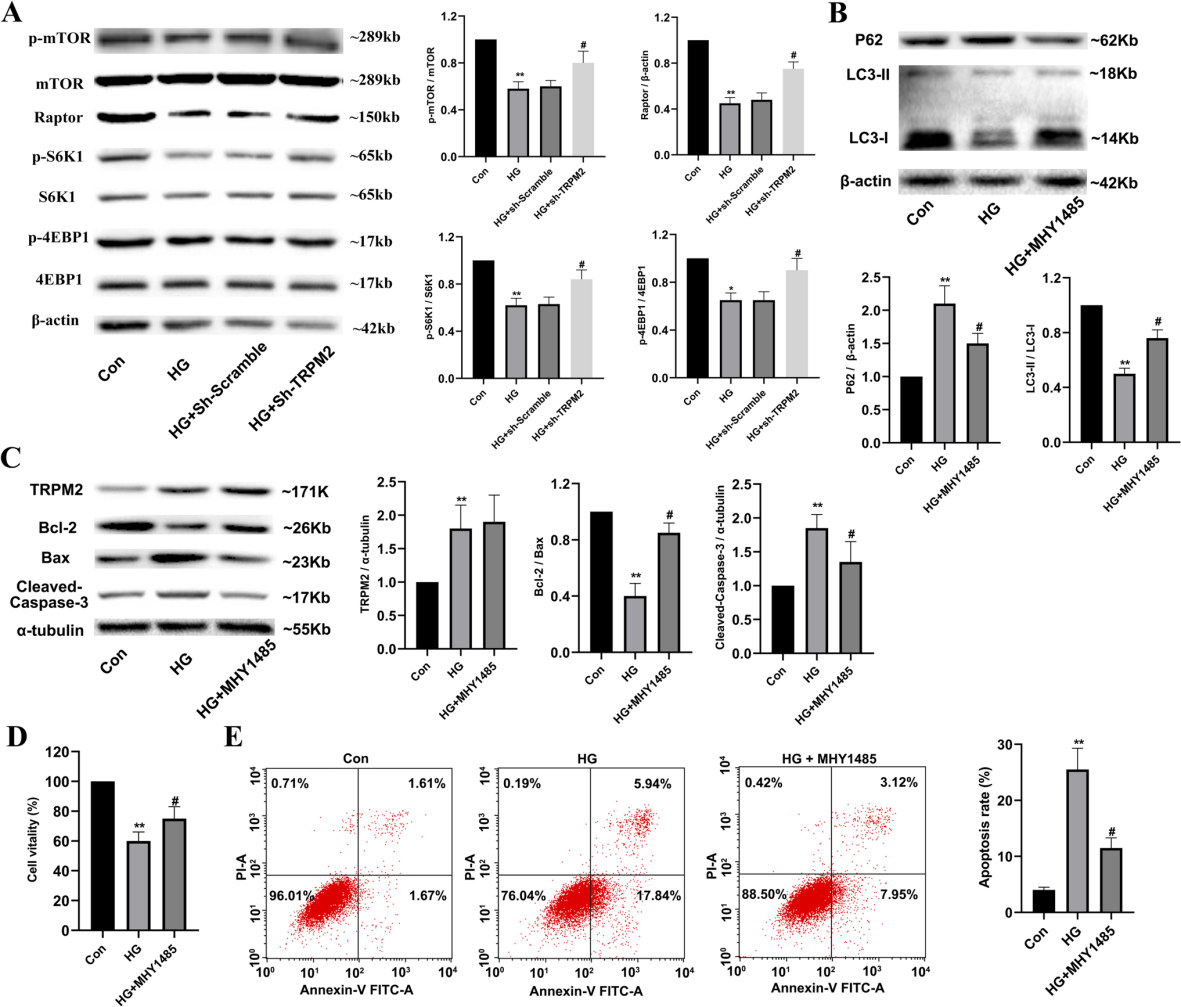
TRPM2 silencing attenuated high glucose-induced apoptosis and autophagy inhibition in cardiomyocytes via regulating the mTORC1 pathway. **A** Representative blots of p-mTOR (Ser2448), Raptor, p-S6K1 (Thr389), and p-4EBP1 (Thr37/46) in high glucose (HG)-stimulated primary cardiomyocytes and corresponding densitometric analysis. β-actin was used as a loading control. Compared to that in controls, the protein expression levels of p-mTOR, Raptor, p-S6K1, and p-4EBP1 were significantly decreased after HG stimulation, which was restored by *TRPM2* silencing. **B** Representative western blots of P62 and LC3 in HG-stimulated cardiomyocytes pre-incubated with MHY1485 and corresponding densitometric analysis. β-actin was used as a loading control. MHY1485 is widely used as an agonist of mTORC1 signaling pathway. NRVMs were treated with 20-µM MHY1485 for one hour to activate the mTORC1 pathway before exposure to 45 mM of HG stimulation for 72 h. Compared to that in controls, the protein expression level of P62 significantly increased; ratio of LC3-II/LC3-I was also significantly decreased, in primary cardiomyocytes after HG stimulation, which was partially reversed by MHY1485 treatment. **C** Representative western blots of TRPM2, Bcl-2, Bax, and cleaved Caspase-3 protein in HG-stimulated cardiomyocytes pre-incubated with MHY1485 and corresponding densitometric analysis. α-tubulin was used as a loading control. Compared to that in controls, the protein expression level of cleaved Caspase-3 significantly increased, the ratio of Bcl-2/Bax significantly decreased, in primary cardiomyocytes after HG stimulation, which was partially restored by MHY1485 treatment. **D** The cell viability was measured by cell counting kit-8 and corresponding statistic analysis. **E** The apoptosis was detected by flow cytometry and corresponding statistic analysis. Compared to that in controls, the cell viability decreased (**D**) and apoptosis increased (**E**) significantly in HG-stimulated cardiomyocytes, which was partially reversed by MHY1485 treatment. The cell experiment was repeated independently for three times. The data are represented as the means ± SE; *P < 0.05, **P < 0.01 versus control group and ^#^*P* < 0.05 versus HG group

Compared to that in controls, the protein expression levels of p-mTOR (Ser2448), Raptor, p-S6K1 (Thr389), and p-4EBP1 (Thr37/46) were significantly decreased after HG stimulation, which was restored by *TRPM2* silencing (Fig. 12A). MHY1485 is widely used as an agonist of mTORC1. Compared to that in controls, the protein expression level of P62 significantly increased, and the ratio of LC3-II/LC3-I was also significantly decreased in primary cardiomyocytes after HG stimulation, which was partially reversed by MHY1485 treatment (Fig. 12B). Compared to that in controls, the protein expression level of cleaved Caspase-3 significantly increased; the ratio of Bcl-2/Bax significantly decreased in primary cardiomyocytes after HG stimulation, which was partially restored by MHY1485 treatment (Fig. 12C). Compared to that in controls, the cell viability decreased (Fig. 12D) and apoptosis increased (Fig. 12E) significantly in cardiomyocytes after HG stimulation, which was also partially reversed by MHY1485 treatment. These results suggested that *TRPM2* silencing might attenuate HG-induced apoptosis and autophagy inhibition in primary cardiomyocytes via regulating the mTORC1 signaling pathway.

## Discussion

Cardiovascular-related complications are responsible for approximately 65% of diabetic death [7]. In the present study, we have successfully established T2DM along with DCM mouse models, which were characterized by glucose metabolic disturbance, myocardial inflammation and fibrosis, cardiomyocyte apoptosis, and autophagy inhibition, resulting in progressive cardiac dysfunction.

TRPM2 is a unique fusion protein with both ion channel function and enzymatic α-kinase activity [10–13]. HFD/STZ-induced diabetic mice exhibited enhanced fluorescence intensity and protein expression of TRPM2 in the hearts as compared to that in controls. To explore whether TRPM2 knockdown improved DCM under the state of hyperglycemia, we knocked down TRPM2 expression with by a single caudal vein injection of ~ 1 × 10^11^ genome copies of recombinant AAV9-U6-shTRPM2 at 6 weeks of age. Noticeably, TRPM2 knockdown improved myocardial systolic and diastolic function by inhibiting apoptosis and promoted autophagy in HFD/STZ-induced diabetic mice. *TRPM2* silencing attenuated HG-induced apoptosis and autophagy inhibition in primary cardiomyocytes via regulating the MEK/ERK mTORC1 signaling pathway.

TRP channels can be activated by oxidative stress-related diseases, such as Alzheimer’s disease, IRI, and diabetes [45]. Hyperglycemia induced an obvious loss of ΔΨm, cytochrome c release, caspase-3 activation, subsequent cardiomyocytes apoptosis, and decreased myocardial contractility [55]. TRPM7 activity or expression would increase in neurons [56], vascular cells [57, 58], and monocytes [59] under the state of hyperglycemia. Activation of TRPM7 channels promotes high glucose-induced endoplasmic reticulum stress and NS20Y neuronal cell apoptosis independently of its kinase activity [56]. Reactive oxygen species (ROS)-TRPM7-ERK1/2 axis plays an important role in hyperglycemia-induced development of the proliferative phenotype in rat aortic vascular smooth muscle cells, where TRPM7 knockdown partially blocked the high glucose-induced phenotype switching [57]. These abnormalities drive the development of DCM. Silencing TRPM7 with siRNA alleviated HG-induced ROS generation and cytotoxicity in Human umbilical vein endothelial cells (HUVECs) involved of ERK pathway [58].

TRPM2-mediated Ca^2+^ entry controls the ROS-induced chemokines responsible for the recruitment of inflammatory cells to sites of injury or infection [23]. TRPM2 functioned by dampening NADPH oxidase-mediated ROS production through depolarization of the plasma membrane in phagocytes, thus inhibited endotoxin-induced lung inflammation in mice [42]. There was an interaction effect between TRPM2-mediated Ca^2+^ entry and p47 phox signaling to induce augmented NADPH oxidase-dependent ROS production and TXNIP-mediated NLRP3 inflammasome activation under high glucose in human monocytic cells [34].

TRPM2-mediated Ca^2+^ influx derived from hyperglycemia-induced ROS triggered lysosomal membrane permeabilization and Zn^2+^-mediated mitochondrial fission in HUVECs [35]. In the pancreatic β-cells, immune cells, and sensory neurons in the brain, TRPM2 interacted with other TRP channels could lead to abnormal insulin secretion or β-cell death depending on the degree of activation [60]. Knockout of TRPM2 impaired incretins-induced insulin secretion and glucose metabolisms in mice [44]. Pharmacological inhibition of TRPM2 reduced hyperglycemia-induced cognitive impairment by down-regulating calcium-related downstream signaling in rats [43]. Diabetes reinforced oxidative stress-induced TRPM2-mediated Ca^2+^ influx and its control by N-acetylcysteine in rat dorsal root ganglion and brain [36]. Melatonin and selenium reduced hyperglycemia-induced excessive ROS release, apoptosis, and Ca^2+^ influx through inhibiting TRPM2 and TRPV1 channel activation in dorsal root ganglion and hippocampus of diabetic rats [38].

TRPM2 channel was also expressed in hippocampus, cortex, and striatum essential for normal cognitive function [61]. Each of the four TRPM2 inhibitors and TRPM2 knockdown by shRNA resulted in obviously reduced neuronal cell death following in vitro oxygen–glucose deprivation only in male neurons [62]. Clotrimazole (a TRPM2 inhibitor) and TRPM2 knockdown distinctly reduced striatal infarcts only in male mice following middle cerebral artery occlusion [62]. Pre-treatment or administration of tat-M2NX (a TRPM2 peptide inhibitor) after reperfusion could alleviate IRI and reduce infarct volume in young adult or aged male mice [63]. TRPM2 knockout inhibited delayed cytosolic Zn^2+^ accumulation, ROS generation, and CA1 pyramidal neuronal death during brain IRI [64]. Gene deficiency of TRPM2 in the peripheral immune system ameliorated neurological outcome and reduced infarct volume in a murine ischemic stroke model [65]. TRPM2 knockout provided neuroprotective effects against brain IRI by inhibiting channel activation [66]. Selenium and resveratrol improved hyperglycemia-induced oxidative retinopathy and optic nerve apoptosis by the down-regulation of TRPM2 activity in mice [67]. These findings above support the targeting of TRPM2 channels as a potential therapeutic strategy to mitigate the pathophysiological progression of oxidative stress-related diseases.

Multiple mechanisms contributed to DCM include the exposure of the heart to hyperglycemia along with increased fatty acids and cytokines [4, 7]. In the diabetic state, pro-inflammatory cytokines induced by hyperglycemia can result in the persistent inflammation in myocardium, which contributes to myocardial dysfunction [52, 68]. Strategies for inhibiting dysregulated inflammation in DCM have attracted increasing attention in recent years. But we found that TRPM2 knockdown did not ameliorate DCM by the regulation of myocardial inflammation. Sustained hyperglycemia leads to a variety of metabolic changes in cardiomyocytes including oxidative stress, which could further trigger apoptotic and autophagic responses [69, 70]. Cell apoptosis is a defining pathological feature of DCM, which leads to progressive cardiac dysfunction [4, 9]. Interestingly, autophagic responses to DCM differ between diabetic types: autophagy was strengthened in T1DM, but was inhibited in T2DM [70]. We found that TRPM2 deficiency improved cardiac dysfunction through inhibiting apoptosis and promoted autophagy in HFD/STZ-induced diabetic mice.

Hyperglycemia increased ROS production and promoted myocardial apoptosis and autophagy via increasing ERK signaling pathway and down-regulating mTORC1 (71). Silencing of High-mobility group box 1 protein (HMGB1) might protect against hyperglycemia-induced cardiomyocyte apoptosis by inhibiting ERK-related signaling pathway [72]. Ginsenoside Rg1 protected diabetic hearts after IRI via HIF-1 α-ERK signaling pathways [73]. Prohibitin overexpression improved DCM by down-regulation of apoptosis through inhibiting ERK1/2 signaling pathway [74]. ROS generation and PARP-1 activation promoted Zn^2+^-induced TRPM2 channel activation, whereby TRPM2-mediated Ca^2+^ influx triggered the PYK2/MEK/ERK signaling pathway as a positive feedback mechanism that amplifies the TRPM2 channel activation, ultimately driving Zn^2+^-induced Ca^2+^ overloading and cell death in microglial cells [75].

mTOR is a serine/threonine-specific protein kinase that contained two different multi-complexes, where mTORC1 is critical in many physiological processes, including cardiovascular diseases [76, 77]. mTORC1 exerted protective effects regulating cardiac apoptosis and autophagy under the state of oxidative stress [76, 77]. Nicorandil inhibited myocardial apoptosis in DCM by activating PI3K/Ak/mTOR signaling pathway [78]. D-Pinitol improved myocardial apoptosis and fibrosis through regulating oxidative stress and PI3K/Akt/mTOR pathways in streptozocin-induced aging-accelerated mice [79]. Exendin-4 and Liraglutide rescued high glucose-induced cardiotoxicity and mitochondrial injury through mTOR/ULK1-dependent autophagy [80]. TRPM2 deficiency provided neuroprotective effects against brain IRI through facilitating autophagy via up-regulating AMPK/mTOR pathway [81]. We found that TRPM2 knockdown improved heart function by inhibiting apoptosis and promoted autophagy via down-regulating MEK/ERK and up-regulating mTORC1 signaling pathway in HFD/STZ-induced diabetic mice.

## Limitation

This study has some limitations. Although the data from animal experiments justified the inclusion of abnormal cytosolic Ca^2+^ handling in cardiomyocytes as an important contributor to DCM [4], we did not record TRPM2-like currents using whole-cell patch-clamp technology. Future research should increase the sample size to eliminate individual differences of in mice. Additionally, considering that TRPM2 knockdown through a single caudal vein injection of recombinant AAV9-U6-shTRPM2 is not fully cardiac specific, future studies should be performed using myocardial specific knockout mice.

## Conclusion

This finding indicated TRPM2 knockdown attenuates hyperglycemia-induced myocardial apoptosis and promotes autophagy in HFD/STZ-induced diabetic mice or HG-stimulated cardiomyocytes via regulating the MEK/ERK and mTORC1 signaling pathway. Understanding the pathophysiological process of DCM is crucial for developing novel therapeutic approaches to prevent symptomatic heart failure, a major cause of morbidity and mortality in diabetic patients. Although future research is needed to clarify the underlying specific mechanism of DCM, TRPM2 could be a promising therapeutic target for DCM therapy.

## Supplementary Information

Below is the link to the electronic supplementary material.

Supplementary file1 (TIF 8961 KB)—Cardiac fibrosis in HFD/STZ-induced diabetic mice. (A) Representative images of collagen matrix deposition in the myocardium according to Masson’s trichrome staining from the different groups (magnification =50x). (B) Corresponding statistic analysis of cardiac fibrosis in A (n=5 per group). (C) α-SMA-positive staining cells in the myocardium from the different groups. (D) Corresponding statistic analysis of α-SMA-positive cells in C (n=5 per group). (E) Representative western blot image of collagen type I in the myocardium from the different groups. α-tubulin was used as a loading control. (F) Corresponding densitometric analysis of blots in E (n = 6 per group). The data are represented as the means ± SE; *P < 0.05 and **P < 0.01

Supplementary file2 (TIF 280 KB)—The mRNA expression of myocardial hypertrophy or fibrosis markers. (A/B) The mRNA expression of ANP and BNP detected by RT-qPCR in the myocardium from the different groups (n=4 per group). (C/D) The mRNA expression of ANP and BNP detected by RT-qPCR in the myocardium from the different groups (n=4 per group). The values were normalized to the housekeeping gene GAPDH. The data are represented as the means ± SE

Supplementary file3 (TIF 13428 KB)—Autophagosomes detected by transmission electron microscopy. (A) Representative transmission electron microscopy images of the left ventricular tissues from the different groups; arrows indicate autophagic vacuoles (n=4 per group). (B) Quantitative analysis of the numbers autophagosomes in different groups. The data are represented as the means ± SE; *P < 0.05

Supplementary file4 (DOC 112 KB)

## Data Availability

The datasets used and/or analyzed during the present study are available from the corresponding author on reasonable request.
