# Correction to: TRPM2 knockdown attenuates myocardial apoptosis and promotes autophagy in HFD/STZ-induced diabetic mice via regulating the MEK/ERK and mTORC1 signaling pathway

**DOI:** 10.1007/s11010-024-05006-z

**Published:** 2024-04-29

**Authors:** Feng Hu, Chaoyang Lin

**Affiliations:** Department of Cardiology, Fujian Medical University Union Hospital, Fuzhou, 350001 Fujian China

**Correction to: Molecular and Cellular Biochemistry** 10.1007/s11010-024-04926-0

Please note the following corrections to this article:

In the Funding (line 3): This study was supported by grants from the science and technology innovation joint fund project of Fujian Provincial Science and Technology Department (2023Y9201).

Instead should say: This study was supported by grants from the science and technology innovation joint fund project of Fujian Provincial Science and Technology Department (2023Y9183).

The original article has been corrected.

