# Supplementary figures and images for: TRPM2 knockdown attenuates myocardial apoptosis and promotes autophagy in HFD/STZ-induced diabetic mice via regulating the MEK/ERK and mTORC1 signaling pathway
Source: Mol Cell Biochem. 2024 Feb 3;479(12):3307–28. doi: 10.1007/s11010-024-04926-0 (PMC11511773; doi:10.1007/s11010-024-04926-0)

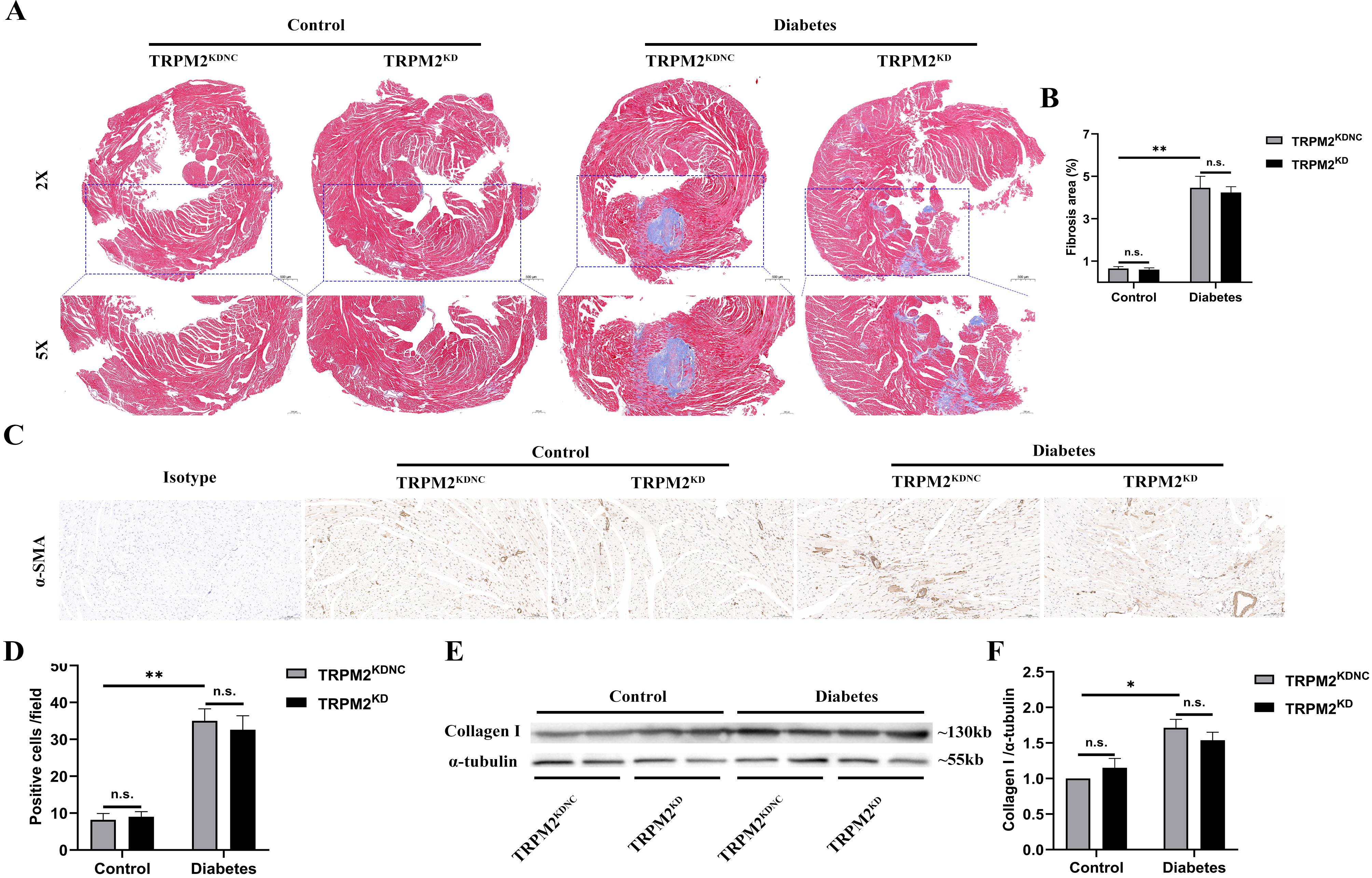

Supplement: Supplementary file 1 — Supplementary file1 (TIF 8961 KB)—Cardiac fibrosis in HFD/STZ-induced diabetic mice. (A) Representative images of collagen matrix deposition in the myocardium according to Masson’s trichrome staining from the different groups (magnification =50x). (B) Corresponding statistic analysis of cardiac fibrosis in A (n=5 per group). (C) α-SMA-positive staining cells in the myocardium from the different groups. (D) Corresponding statistic analysis of α-SMA-positive cells in C (n=5 per group). (E) Representative western blot image of collagen type I in the myocardium from the different groups. α-tubulin was used as a loading control. (F) Corresponding densitometric analysis of blots in E (n = 6 per group). The data are represented as the means ± SE; *P < 0.05 and **P < 0.01 [file 11010_2024_4926_MOESM1_ESM.tif]

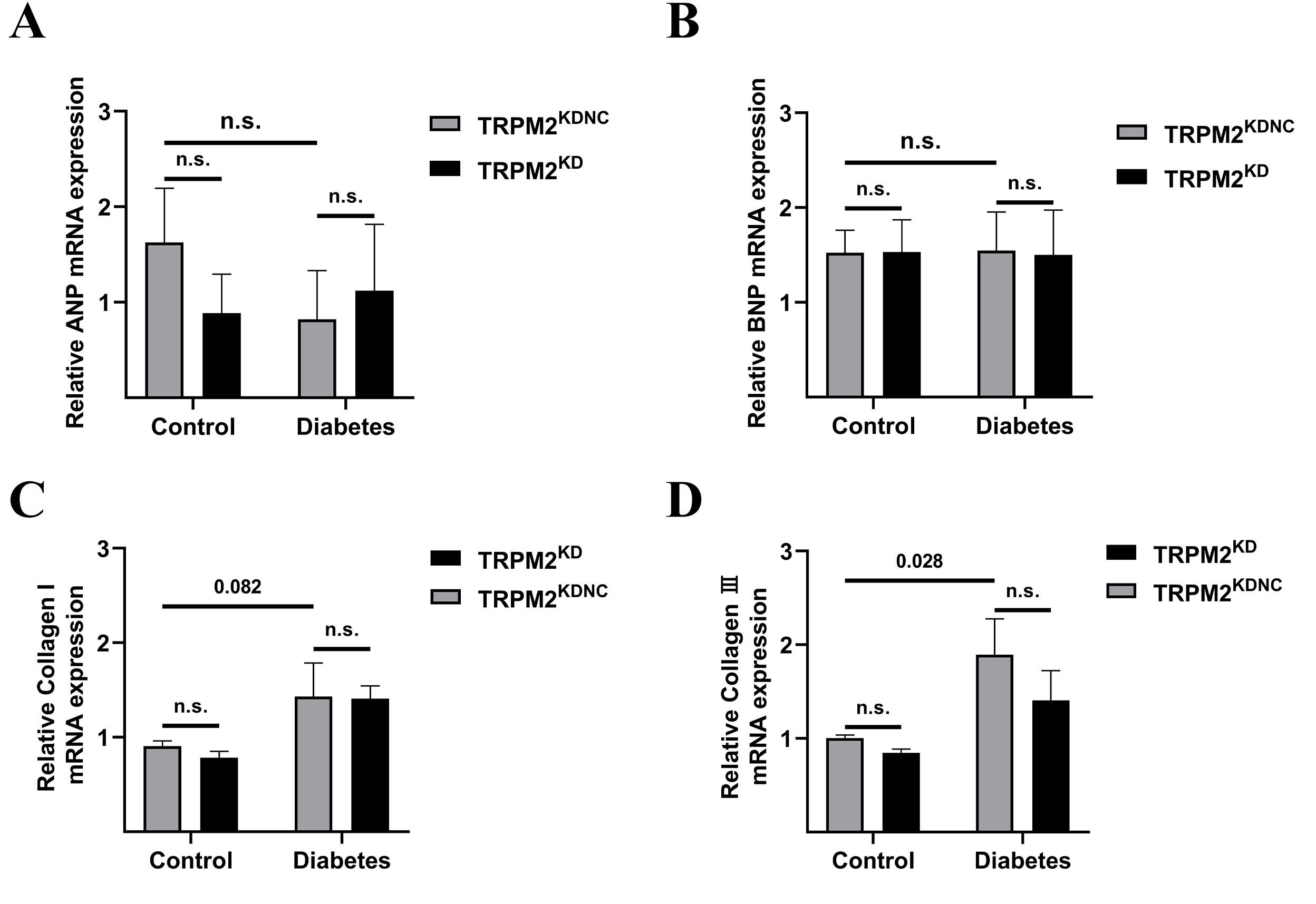

Supplement: Supplementary file 2 — Supplementary file2 (TIF 280 KB)—The mRNA expression of myocardial hypertrophy or fibrosis markers. (A/B) The mRNA expression of ANP and BNP detected by RT-qPCR in the myocardium from the different groups (n=4 per group). (C/D) The mRNA expression of ANP and BNP detected by RT-qPCR in the myocardium from the different groups (n=4 per group). The values were normalized to the housekeeping gene GAPDH. The data are represented as the means ± SE [file 11010_2024_4926_MOESM2_ESM.tif]

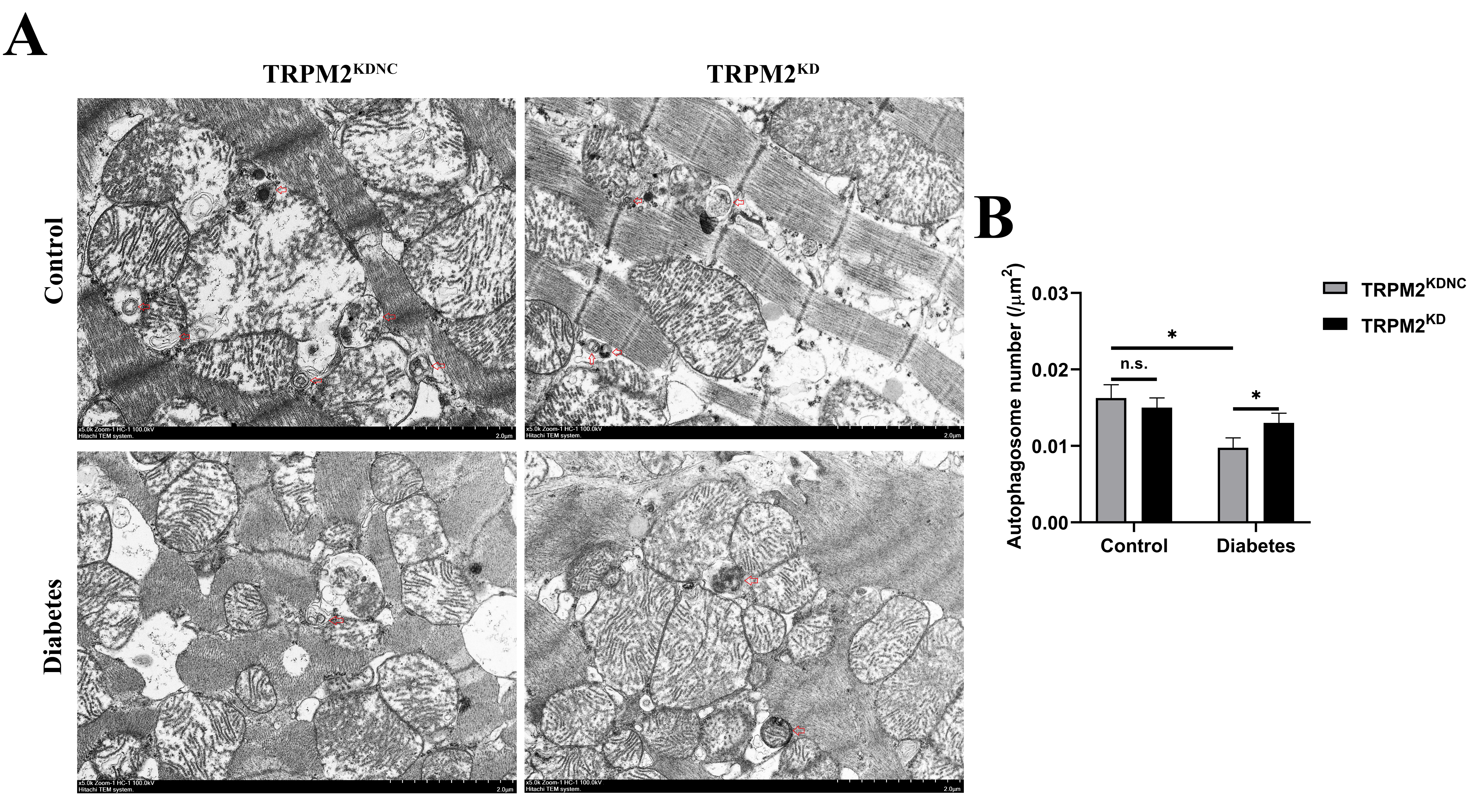

Supplement: Supplementary file 3 — Supplementary file3 (TIF 13428 KB)—Autophagosomes detected by transmission electron microscopy. (A) Representative transmission electron microscopy images of the left ventricular tissues from the different groups; arrows indicate autophagic vacuoles (n=4 per group). (B) Quantitative analysis of the numbers autophagosomes in different groups. The data are represented as the means ± SE; *P < 0.05 [file 11010_2024_4926_MOESM3_ESM.tif]
