# Supplementary material for: TRPM2 knockdown attenuates myocardial apoptosis and promotes autophagy in HFD/STZ-induced diabetic mice via regulating the MEK/ERK and mTORC1 signaling pathway
Source: Mol Cell Biochem. 2024 Feb 3;479(12):3307–28. doi: 10.1007/s11010-024-04926-0 (PMC11511773; doi:10.1007/s11010-024-04926-0)
Supplement: Supplementary file 4 — Supplementary file4 (DOC 112 KB) [file 11010_2024_4926_MOESM4_ESM.doc]

**Supplementary Table Legends**

**Supplementary Table 1: Primer sequence for real time quantitative PCR (RT-qPCR).**

| **Gene name** | **Primer sequences** |
| --- | --- |
| ANP | F: 5’-GCTTCCAGGCCATATTGGAG-3’  R: 5’-GGGGGCATGACCTCATCTT-3’ |
| BNP | F: 5’-GAGGTCACTCCTATCCTCTGG-3’  R: 5’-GCCATTTCCTCCGACTTTTCTC-3’ |
| Collagen I | F: 5’-GCTCCTCTTAGGGGCCACT-3’ |
|  | R: 5’-ATTGGGGACCCTTAGGCCAT-3’ |
| Collagen Ⅲ | F: 5’-CTGTAACATGGAAACTGGGGAAA-3’ |
|  | R: 5’-CCATAGCTGAACTGAAAACCACC-3’ |
| GAPDH | F: 5’-AGGTCGGTGTGAACGGATTTG-3’ |
|  | R: 5’-GGGGTCGTTGATGGCAACA-3’ |

**Supplementary Table 2: List of primary antibodies for** **Western blot.**

| **Name** | **Catalog** | **Dilutions** | **Company** |
| --- | --- | --- | --- |
| TRPM2 | #PA1-46473 | 1:1000 | Invitrogen |
| Cleaved Caspase-3 | #ab214430 | 1:1000 | Abcam |
| Caspase 1 | #A0964 | 1:1000 | ABclonal |
| Bcl-2 | #ab196495 | 1:1000 | Abcam |
| Bax | #ab32503 | 1:1000 | Abcam |
| Collagen I | #ab260043 | 1:100 | Abcam |
| P62 | #ab109012 | 1:10000 | Abcam |
| ULK1 | #20986-1-AP | 1:1000 | Proteintech |
| LC3B | #ab192890 | 1:1000 | Abcam |
| Phospho-MEK1/2 (Ser217/221) | #9154 | 1:1000 | CST |
| MEK1/2 | #4694 | 1:1000 | CST |
| Phospho-ERK1/2 (Thr202/Tyr204) | #4370 | 1:2000 | CST |
| ERK1/2 | #4695 | 1:1000 | CST |
| Phospho-mTOR (Ser2448) | #ab109268 | 1:1000 | Abcam |
| mTOR | #ab32028 | 1:1000 | Abcam |
| Phospho-S6K1 (Thr389) | #9206 | 1:1000 | CST |
| S6K1 | #2708 | 1:1000 | CST |
| Phospho-4EBP1 (Thr37/46) | #2855 | 1:1000 | CST |
| 4EBP1 | #9644 | 1:1000 | CST |
| Raptor | #2280 | 1:1000 | CST |
| GAPDH | #60004 | 1:10000 | Proteintech |
| β-actin | #ab6276 | 1:5000 | Abcam |
| α-tubulin | #11224-1-AP | 1:5000 | Proteintech |

| **Supplementary Table 3: Dynamic change of fasting blood glucose (FBG) in each group.** | | | | | | | |
| --- | --- | --- | --- | --- | --- | --- | --- |
| FBG（mmol/L） | 0 week | 4 week | 8 week | 12 week | 16 week | 20 week | 24 week |
| TRPM2^KDNC^; Control |  |  |  |  |  |  |  |
| Mean | 6.42 | 6.63 | 6.43 | 6.53 | 6.48 | 6.48 | 6.50 |
| Standard error | 0.15 | 0.16 | 0.18 | 0.14 | 0.15 | 0.16 | 0.16 |
| TRPM2^KD^; Control |  |  |  |  |  |  |  |
| Mean | 6.60 | 6.78 | 6.37 | 6.63 | 6.67 | 6.5 | 6.65 |
| Standard error | 0.17 | 0.13 | 0.13 | 0.16 | 0.15 | 0.23 | 0.13 |
| TRPM2^KDNC^; Diabetes |  |  |  |  |  |  |  |
| Mean | 6.75 | 6.77 | 18.28 | 19.32 | 20.87 | 21.52 | 22.50 |
| Standard error | 0.14 | 0.17 | 1.13 | 0.82 | 0.86 | 0.68 | 0.69 |
| TRPM2^KD^; Diabetes |  |  |  |  |  |  |  |
| Mean | 6.58 | 6.60 | 17.32 | 18.73 | 20.28 | 21.13 | 22.43 |
| Standard error | 0.15 | 0.20 | 1.03 | 0.81 | 0.51 | 0.59 | 0.20 |
| *P1* | 0.400 | 0.529 | 0.952 | 0.905 | 0.801 | 0.980 | 0.780 |
| *P2* | 0.134 | 0.576 | <0.001 | <0.001 | <0.001 | <0.001 | <0.001 |
| *P3* | 0.444 | 0.485 | 0.386 | 0.490 | 0.427 | 0.573 | 0.901 |
| The data are shown as the mean ± SD, n= 6 per group; *P1* represented TRPM2^KDNC^; Control versus TRPM2^KD^; Control. *P2* represented TRPM2^KDNC^; Control versus TRPM2^KDNC^; Diabetes. *P3* represented TRPM2^KDNC^; Diabetes versus TRPM2^KD^; Diabetes. | | | | | | | |

| **Supplementary Table 4: Dynamic change of body weight in each group.** | | | | | | | | | | | | | |
| --- | --- | --- | --- | --- | --- | --- | --- | --- | --- | --- | --- | --- | --- |
| Body weight（g） | 0 week | 2 week | 4 week | 6 week | 8 week | 10 week | 12 week | 14 week | 16 week | 18 week | 20 week | 22 week | 24 week |
| TRPM2^KDNC^; Control |  |  |  |  |  |  |  |  |  |  |  |  |  |
| Mean | 22.27 | 23.77 | 26.07 | 27.05 | 27.53 | 28.85 | 29.33 | 29.95 | 30.93 | 31.50 | 31.83 | 32.22 | 33.45 |
| Standard error | 0.25 | 0.28 | 0.34 | 0.49 | 0.51 | 0.50 | 0.52 | 0.48 | 0.45 | 0.42 | 0.43 | 0.44 | 0.52 |
| TRPM2^KD^; Control |  |  |  |  |  |  |  |  |  |  |  |  |  |
| Mean | 22.12 | 24.10 | 26.02 | 26.90 | 27.82 | 29.28 | 29.80 | 30.78 | 31.65 | 31.93 | 32.38 | 33.05 | 34.28 |
| Standard error | 0.25 | 0.43 | 0.38 | 0.39 | 0.38 | 0.41 | 0.40 | 0.44 | 0.43 | 0.41 | 0.42 | 0.42 | 0.42 |
| TRPM2^KDNC^; Diabetes |  |  |  |  |  |  |  |  |  |  |  |  |  |
| Mean | 22.10 | 23.70 | 25.53 | 26.35 | 26.98 | 28.35 | 29.23 | 29.85 | 30.77 | 31.15 | 31.42 | 31.87 | 32.53 |
| Standard error | 0.20 | 0.42 | 0.32 | 0.31 | 0.29 | 0.25 | 0.34 | 0.32 | 0.19 | 0.26 | 0.27 | 0.27 | 0.23 |
| TRPM2^KD^; Diabetes |  |  |  |  |  |  |  |  |  |  |  |  |  |
| Mean | 22.03 | 23.73 | 25.70 | 26.43 | 26.90 | 28.33 | 29.02 | 29.45 | 30.82 | 31.18 | 31.63 | 31.90 | 32.75 |
| Standard error | 0.23 | 0.22 | 0.26 | 0.24 | 0.26 | 0.19 | 0.19 | 0.22 | 0.19 | 0.22 | 0.19 | 0.19 | 0.56 |
| *P1* | 0.651 | 0.509 | 0.915 | 0.777 | 0.599 | 0.409 | 0.394 | 0.135 | 0.150 | 0.378 | 0.267 | 0.106 | 0.110 |
| *P2* | 0.615 | 0.894 | 0.263 | 0.196 | 0.312 | 0.342 | 0.854 | 0.854 | 0.731 | 0.475 | 0.398 | 0.486 | 0.081 |
| *P3* | 0.840 | 0.947 | 0.723 | 0.875 | 0.877 | 0.974 | 0.690 | 0.463 | 0.918 | 0.945 | 0.658 | 0.947 | 0.669 |
| The data are shown as the mean ± SD, n= 6 per group; *P1* represented TRPM2^KDNC^; Control versus TRPM2^KD^; Control. *P2* represented TRPM2^KDNC^; Control versus TRPM2^KDNC^; Diabetes. *P3* represented TRPM2^KDNC^; Diabetes versus TRPM2^KD^; Diabetes. | | | | | | | | | | | | | |
